# At Short Telomeres Tel1 Directs Early Replication and Phosphorylates Rif1

**DOI:** 10.1371/journal.pgen.1004691

**Published:** 2014-10-16

**Authors:** Akila Sridhar, Sylwia Kedziora, Anne D. Donaldson

**Affiliations:** Institute of Medical Sciences, University of Aberdeen, Foresterhill, Aberdeen, Scotland, United Kingdom; Rutgers New Jersey Medical School,, United States of America

## Abstract

The replication time of *Saccharomyces cerevisiae* telomeres responds to TG_1–3_ repeat length, with telomeres of normal length replicating late during S phase and short telomeres replicating early. Here we show that Tel1 kinase, which is recruited to short telomeres, specifies their early replication, because we find a *tel1*Δ mutant has short telomeres that nonetheless replicate late. Consistent with a role for Tel1 in driving early telomere replication, initiation at a replication origin close to an induced short telomere was reduced in *tel1*Δ cells, in an S phase blocked by hydroxyurea. The telomeric chromatin component Rif1 mediates late replication of normal telomeres and is a potential substrate of Tel1 phosphorylation, so we tested whether Tel1 directs early replication of short telomeres by inactivating Rif1. A strain lacking both Rif1 and Tel1 behaves like a *rif1*Δ mutant by replicating its telomeres early, implying that Tel1 can counteract the delaying effect of Rif1 to control telomere replication time. Proteomic analyses reveals that in *yku70*Δ cells that have short telomeres, Rif1 is phosphorylated at Tel1 consensus sequences (S/TQ sites), with phosphorylation of Serine-1308 being completely dependent on Tel1. Replication timing analysis of a strain mutated at these phosphorylation sites, however, suggested that Tel1-mediated phosphorylation of Rif1 is not the sole mechanism of replication timing control at telomeres. Overall, our results reveal two new functions of Tel1 at shortened telomeres: phosphorylation of Rif1, and specification of early replication by counteracting the Rif1-mediated delay in initiation at nearby replication origins.

## Author Summary

The ends of chromosomes are protected by specialized structures called telomeres, which prevent their recognition as DNA breaks and enable recruitment of telomerase, the reverse transcriptase that maintains telomere length by replacing terminal TG-repeat sequences lost during successive rounds of DNA replication. Chromosomal DNA is replicated from initiation sites called origins, which are activated in a reproducible temporal sequence. Replication origins close to telomeres are subject to specialized temporal control that contributes to telomere stabilization: origins close to normal-length telomeres initiate replication late, while those close to shortened telomeres initiate early. Here we uncover the control mechanism that links telomere length with replication timing. Rif1, one of the components of telomeric chromatin, directs late replication of normal telomeres by delaying the activation of nearby origins. Our experiments show that a kinase called Tel1, which is recruited to shortened telomeres, neutralizes the origin-delaying activity of Rif1. We also find that Tel1 phosphorylates Rif1 at short telomeres, although our investigation shows this phosphorylation is not the sole mechanism through which Tel1 prevents Rif1-mediated replication delay. Since correct telomere replication timing control is important for telomerase-mediated length maintenance, this discovery represents an important step towards understanding the molecular mechanisms that ensure proper long-term stabilization of chromosome ends, as well as the controls over the DNA replication temporal program.

## Introduction

Chromosomal DNA replication occurs according to a regulated program, with some replication origins initiating early and others late in S phase [1], [2]. *S. cerevisiae* telomeres provide a good model for understanding molecular controls over the temporal regulation of DNA replication. The replication time of *S. cerevisiae* telomeric regions is regulated by telomere length; chromosome regions close to telomeres with a normal length terminal TG_1–3_ tract generally replicate late, but those close to telomeres with a shortened TG_1–3_ tract replicate early [3], [4]. This control is mediated through altered initiation time of replication origins. Telomeres may be replicated either by replication forks from an origin within the subtelomeric repeat sequences (X or Y′ ARS elements), or by a fork arriving from a nearby telomere-proximal origin (such as ARS522, close to chromosome V-right; previously known as ARS501) [5]–[7]. Normal length telomeres can direct the late activation of such origins, while telomeric and telomere-proximal origins activate earlier if next to a shortened telomere—as demonstrated by experiments using recombination-based excision of TG_1–3_ repeats or the mutation *yku70*Δ that causes shortened telomeres [3], [4], [8]. Telomere repeat length can affect origins up to 40 kb from the chromosome end [4]. Earlier replication is proposed to favor telomerase recruitment and TG_1–3_ repeat lengthening [9]–[11]. However, how cells detect and respond to telomere length in order to control the replication time of telomeres remains unclear.

The end-replication problem causes shortening of terminal TG_1–3_ tracts in successive cell cycles, and a network of controls detects critically short telomeres and ensures they are preferentially elongated by telomerase enzyme [12]. The mechanisms that detect TG_1–3_ tract length to control replication timing are likely to overlap with mechanisms that ensure preferential elongation of short telomeres. Indeed, the Rif1 protein is already implicated in both pathways. *S. cerevisiae* Rif1 binds to the TG_1–3_ repeat recognition factor Rap1 and with Rif2 regulates telomerase recruitment in response to telomere length [13], [14]. Rif1 and Rif2 appear to ‘count’ the telomeric repeats and repress telomerase recruitment if the TG_1–3_ tract does not require extension. Cells lacking either Rif1 or Rif2 have abnormally long telomeres due to uncontrolled lengthening by telomerase [13]. The molecular mechanism by which Rif1 represses telomerase recruitment is still under investigation. Long and short telomeres bind similar amounts of Rif1 [15]; one proposal is that molecular modifications occurring selectively at short telomeres may relieve the repressive effect of Rif1 on telomerase recruitment [16]. For Rif2, number of molecules may determine the repressive effect on telomerase, since more Rif2 molecules are present at long than short telomeres [17].

As well as acting in the pathway that recognizes short telomeres for lengthening, Rif1 is involved in controlling telomere replication time in response to length [4]. Specifically, in cells lacking Rif1 the link between telomere length and replication time is broken, since the telomeres of a *rif1*Δ mutant replicate early despite being abnormally long. Recently, Rif1 has been implicated as a regulator of replication timing more generally, having a repressive effect on genome-wide DNA replication mediated through recruitment of Protein Phosphatase 1 [18]–[23].

Tel1, a PIK (phosphatidylinositol 3-kinase)-related checkpoint kinase, is involved in short telomere recognition. Tel1 binds to short telomeres and contributes to their preferential recruitment of telomerase [15], [24]–[26]. Tel1 is recruited by interacting with the C-terminus of Xrs2, a subunit of the MRX (Mre11-Rad50-Xrs2) nuclease complex, which is also enriched at short telomeres. The kinase activity of Tel1 is required for its role in telomere maintenance [27]. Potential targets for Tel1 phosphorylation at telomeres include Xrs2, Mre11 [28] and the telomeric single-stranded binding protein Cdc13 [29], but it is unclear whether phosphorylation of these targets is important for telomere lengthening [30]; discussed in [16]. There is some overlap in function between Tel1 and Mec1, the other yeast PIK checkpoint kinase, but while telomeres in *tel1*Δ mutant cells are extremely short, lack of Mec1 causes only a mild telomere length defect [31], [32]. In general, Tel1 seems to play the primary role in regulating telomere function while Mec1 is the major checkpoint kinase.

Since Tel1 is preferentially recruited to short telomeres, we investigated whether Tel1 is also involved in the pathway that detects short telomeres to specify early replication. We show here that Tel1 is required to drive the early replication of short telomeres, and that it acts upstream of Rif1 in the pathway that controls telomere replication timing. We tested whether Tel1 phosphorylates Rif1, and identified two SQ (i.e. Tel1 consensus) sites that are preferentially phosphorylated in a short telomere mutant. Phosphorylation of one of the sites, Serine-1308, is completely dependent on the presence of Tel1. Mutation of these sites did not prevent the early replication of short telomeres, suggesting that Rif1 phosphorylation is not the sole mechanism through which Tel1 drives early replication. Our results are consistent with a model in which Tel1 that is recruited to short telomeres counteracts the repressive effect of Rif1 on replication initiation at nearby origins, to promote early origin activation and advance the replication time of short telomeres.

## Results

### Tel1 is required for the early replication of shortened telomeres

**Figure 1:**
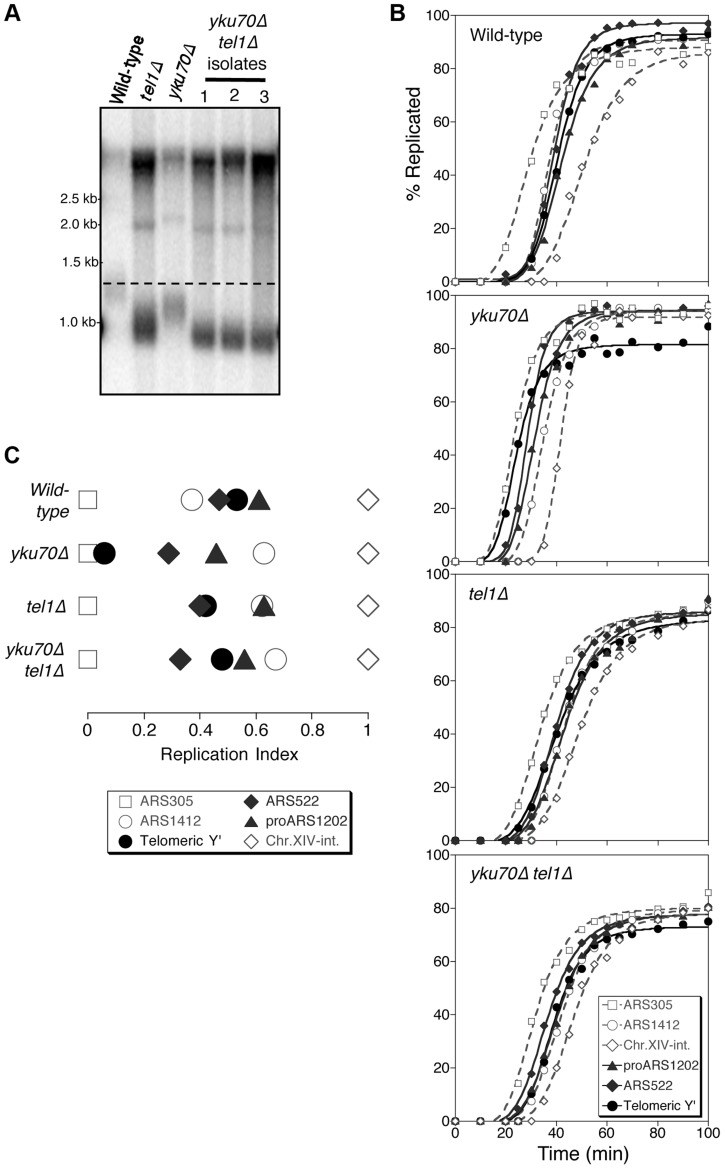
Tel1 is required for early replication of short telomeres. (A) Telomere length analysis in wild-type (*YKU70 TEL1*), *tel1*Δ, *yku70*Δ, and *yku70*Δ* tel1*Δ strains. Terminal chromosome fragments were detected by probing a Southern blot of XhoI-digested genomic DNA for TG_1–3_ sequence. Smear represents average length of Y′ telomeres. (B) Replication kinetics of various genomic sequences in wild-type and short telomere mutants *yku70*Δ, *tel1*Δ and *yku70*Δ* tel1*Δ. Telomere-proximal sequences shown are Y′ (solid line with filled circles), ARS522 (solid line with filled diamonds), and proARS1202 (solid line with filled triangles). Non-telomeric marker sequences (dashed lines) are early origins ARS305 (open squares), late origin ARS1412 (open circles), and Chr XIV-internal sequences (open diamonds). Strains were released from α-factor block at 30°C. (C) Replication indices (RI) values from experiments in B, where replication times are normalized to early origin ARS305 (RI = 0) and Chr.XIV-int (RI = 1). Strains are BB14-3a (wild-type), ASY5 (*tel1*Δ), AW99 (*yku70*Δ) and ASY13 (*yku70*Δ* tel1*Δ; corresponding to second isolate in part A); all are in A364a background as listed in Table S1.

To investigate the mechanism linking telomere length with replication timing, we examined the role of Tel1, since this kinase is implicated in telomere length detection. *tel1*Δ cells have very short telomeres as shown in Fig. 1A, due to defective telomerase recruitment. If telomere replication time is still correctly coupled to TG_1–3_ tract length in this mutant, we would expect the short telomeres of a *tel1*Δ strain to replicate early—like the telomeres of a *yku70*Δ mutant, which replicate earlier than normal because they are short [4].

Replication time can be measured using the dense isotope transfer method, in which cells blocked in G1 phase with α-factor are transferred from isotopically dense to light medium. Upon release into S phase the transition of specific sequences from heavy∶heavy to heavy∶light DNA fractions on cesium gradient centrifugation is then monitored. Replication kinetics of particular sequences are plotted (Fig. 1B), and replication time assigned as the time at which half the final level of replication has occurred. Since kinetics of α-factor release show some variability between experiments, the replication program can be usefully summarized using ‘replication indices’ (Fig. 1C), with the various replication times normalized to early and late-replicating marker sequences (ARS305 and Chr XIV-int respectively) [33]. Replication times plotted relative to ARS305 are shown in Fig. S1.

In wild-type cells, the subtelomeric Y′ repeat sequences (indicative of average telomeric replication) replicate late in S phase (Fig. 1B; top panel, solid line with filled circles, Fig. 1C&S1, filled circle), 3.4 min later than the internal late replication origin ARS1412 [33], [34]. In the *yku70*Δ mutant that has shortened TG_1–3_ repeat sequences, the Y′ sequences replicate much earlier, at a similar time to early origin ARS305 (Fig. 1B,C&S1) [4], [8]. Examining replication kinetics in a *tel1*Δ mutant strain revealed that Y′ sequences replicated late, close to their normal replication time (Fig. 1B; third panel from top & Fig. 1C). Telomere-proximal sequences (ARS522 and proARS1202) show a similar trend (solid curve with filled diamonds and solid curve with filled triangles, respectively; Fig. 1B), so that overall the replication program of the *tel1*Δ mutant resembles that of wild-type cells (Fig. 1C). Since the *tel1*Δ mutant has very short telomeres (even shorter than those of *yku70*Δ; Fig. 1A) this result suggests that in the absence of Tel1 kinase, the replication time of telomeric regions is uncoupled from telomere length.

Telomeres of a *yku70*Δ mutant are short due to defects in telomere capping and extension. *yku70*Δ cells can detect telomere length status—since *yku70*Δ telomeres replicate early and Tel1 is correctly recruited to the short telomeres of a *yku70*Δ strain [35]. Early telomere replication in a *yku70*Δ mutant appears to result from telomere shortness, since restoring telomeres to wild-type length in a *yku70*Δ background leads to recovery of normal, late telomere replication [4]. The *yku70*Δ mutant therefore provides a convenient tool to investigate the controls linking telomere length to replication control. Note that the strength of the effect on telomere length of the *yku70*Δ mutation differs in A364a (used for timing replication in this study) and BY4741 yeast strain backgrounds (Fig. S2). Strain dependence of the effect of *yku70*Δ on telomere length was previously observed (compare [36], [37] with [38]–[40]). The reason for the strain dependence is not known, but the effects of the *yku70*Δ mutation on replication timing appear similar regardless of whether the effect on telomere length is weak or strong [8], [41], [42].

To understand whether Tel1 is required to transmit the signal for the early replication of short telomeres, we examined the replication program of a *yku70Δ tel1*Δ double mutant. This mutant has extremely short telomeres similar to a *tel1*Δ mutant (Fig. 1A), but subtelomeric (Y′) and telomere-proximal (ARS522 and proARS1202) sequences replicated later than in *yku70*Δ single mutant, with replication timing similar to that in wild-type or *tel1*Δ cells (Fig. 1B,C&S1). While precise replication times and order show some variability between experiments [33], repetition of these experiments confirmed the general trends (Fig. S3). Overall, these results suggest that in the absence of Tel1, *yku70*Δ telomeres are no longer sensed as short and hence not replicated early, implying that Tel1 is involved in specifying early replication of short telomeres.

### Tel1 stimulates the early initiation of a replication origin next to an induced short telomere

**Figure 2:**
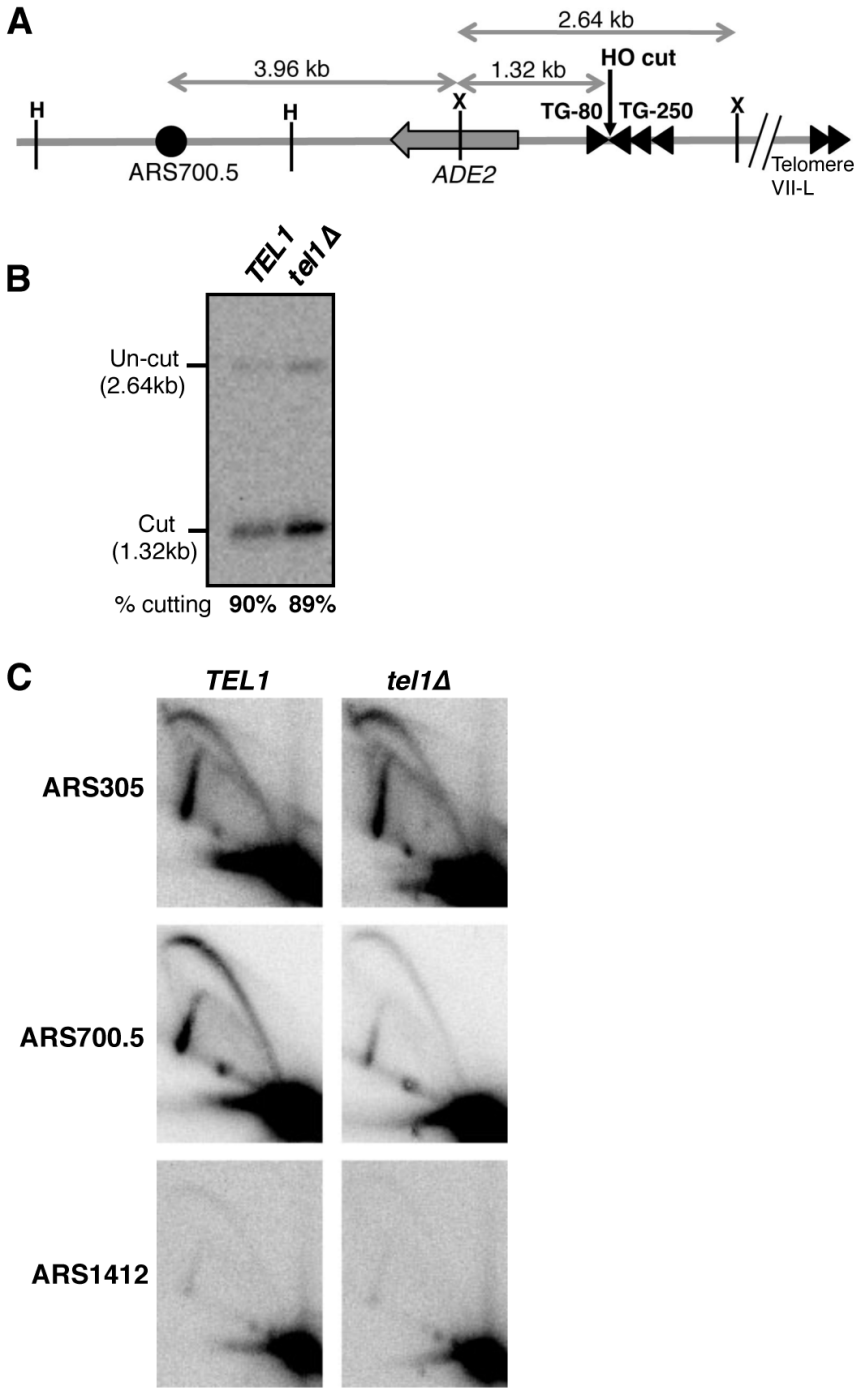
Tel1 stimulates activation of an origin neighboring an induced short telomere upon release into hydroxyurea. (A) Cartoon showing HO endonuclease-inducible short telomere construct on the left arm of Chr. VII, with positions of HindIII (H) and XmnI (X) restriction sites. Triangles represent TG repeat sequences and the filled circle, ARS700.5. Not to scale. (B) HO endonuclease cutting efficiency in the hydroxyurea-arrested cultures used for 2D gel analysis in C. Cells were arrested with α-factor then galactose added to induce HO cutting, followed by release into hydroxyurea. (C) 2D gel analysis of replication intermediates present at early origin ARS305 (upper panels), ARS700.5 (middle panels), and late origin ARS1412 (lower panels), in *TEL1* (left) and *tel1*Δ (right) strains. The same blot of HindIII-digested DNA was probed sequentially for the three origins. Strains used are YAB1410, SMKY10 (*TEL1*) and SMKY13 (*tel1*Δ).

We cannot exclude the possibility that effects shown above result from mutant phenotypes unrelated to telomere length. For example, the effect on replication timing of telomere uncapping in *yku70*Δ has not been tested. We therefore examined whether Tel1 promotes early telomere replication using an alternative mode of telomere shortening. We utilized a yeast strain in which a short telomere can be created by induction of HO endonuclease in cells blocked in G1 phase, as illustrated in Fig. 2A and similar to the construct previously described [43]. In this system, an HO cut site close to the left end of chromosome VII is flanked by short (80 bp) and long (250 bp) TG_1–3_ tracts on its centromere- and telomere-proximal sides respectively. Cutting with HO endonuclease in G1-blocked cells creates a single shortened telomere which, following release into S phase, stimulates earlier initiation at the neighboring, normally late-replicating origin ARS700.5. ARS700.5 is located 18 kb from unmodified telomere VII-left and lies 5.3 kb from the HO cut site in this construct [Cooley & Bianchi, personal communication]. In a small-scale experiment we found that HO cutting levels exceeded 67% 5.5 hr after galactose addition, confirming that short telomere induction occurred in the majority of cells (Fig. S4A).

When *S. cerevisiae* cells attempt S phase in the presence of the replication inhibitor hydroxyurea (HU), early origins are activated but late origin initiation is repressed by the Rad53-mediated S phase checkpoint [44]–[46]. Two-dimensional gel analysis of origin activation levels [47] after release into hydroxyurea therefore provides a proxy for differences in origin initiation time.

The short telomere was induced by HO cutting and cells were then released into HU-containing medium (during which cutting levels reached 90%; Fig. 2B). At the control early-initiating replication origin ARS305, 2-dimensional gel analysis revealed strong bubble arcs in both *TEL1* and *tel1*Δ strains (Fig. 2C, upper panels). In contrast only low levels of replication intermediates were observed at the control late origin ARS1412 (Fig. 2C, lower panels), due to checkpoint-mediated late origin repression. At ARS700.5 close to the induced short telomere, a strong bubble arc was observed in the *TEL1* strain, consistent with stimulation of early ARS700.5 initiation as expected. Bubble arc intensity was however substantially reduced in the *tel1*Δ mutant (Fig. 2C, middle panels), revealing that Tel1 is needed to drive early, checkpoint-resistant initiation of ARS700.5 following nearby short telomere induction. Quantitation of the bubble arc signal (as shown in Fig. S4B&C) revealed 4.8-fold-reduction in bubble arc intensity at ARS700.5 in the *tel1*Δ strain. In a construct with ARS700.5 placed proximal to a long telomere repeat a bubble arc was almost undetectable (Fig. S5), confirming that early activation of this origin depends on the nearby induced short telomere.

Our 2-dimensional gel analysis therefore confirmed that after nearby short telomere induction, the absence of Tel1 changes the character of ARS700.5 from that of an early-initiating origin to that of a late replication origin. The results were therefore consistent with the replication timing analyses in Figs. 1 & S3, showing that Tel1 is required to specify early replication of chromosomal regions in proximity to a short telomere.

We also attempted to use isotope labeling-based replication timing analysis to examine ARS700.5 replication following short telomere induction, but inefficient and variable HO cutting after growth in the minimal medium required for this technique prevented satisfactory analysis of replication timing.

### Tel1 acts upstream of Rif1 in controlling telomere replication timing

**Figure 3:**
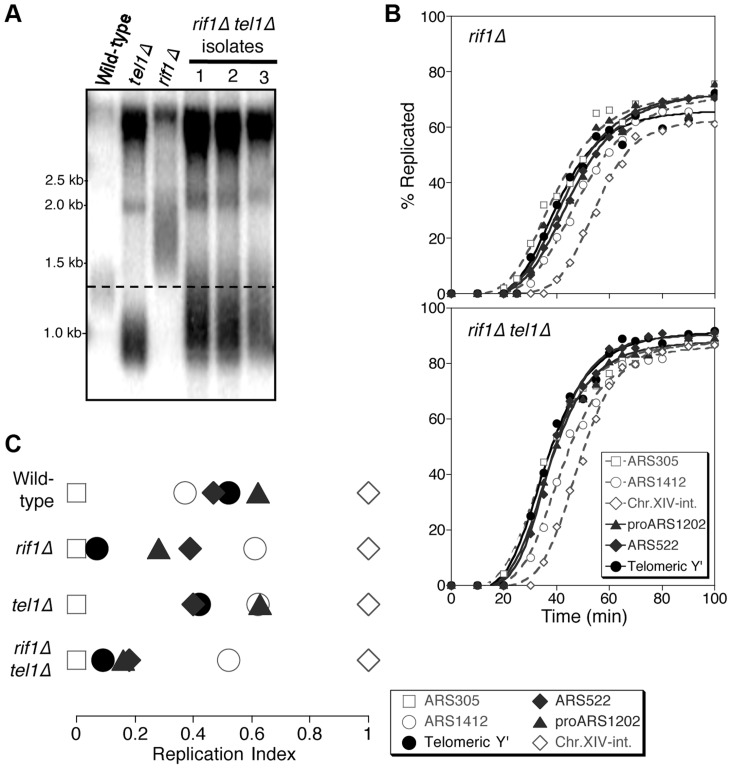
Rif1 acts downstream of Tel1 in regulating telomere replication time. (A) Telomere length analysis in wild-type, *tel1*Δ, *rif1*Δ and *rif1*Δ* tel1*Δ strains. Southern blot analysis carried out as in Fig. 1A. (B) Replication kinetics of various genomic sequences in *rif1*Δ and *rif1*Δ* tel1*Δ strains. Plots and symbols as in Fig. 1B. (C) Replication indices from experiments in B, along with values from wild-type and *tel1*Δ experiments from Fig. 1. Strains are HYLS44 (*rif1*Δ) and ASY14 (*rif1*Δ* tel1*Δ; corresponding to first isolate in part A).

Rif1 is implicated in the control of replication timing in response to telomere length, since in a *rif1*Δ mutant the link between telomere length and replication time is uncoupled. Specifically, in a *rif1*Δ mutant the TG_1–3_ tracts are over-extended (Fig. 3A), but cells fail to detect the length of their telomeres and replicate them inappropriately early (Fig. 3B,C & Fig. S6A) [4]. Consistently, ARS700.5 initiates prior to the S phase checkpoint in a *rif1*Δ mutant with an induced short telomere (Fig. S6B). Early replication of the long *rif1*Δ telomeres presents an interesting reversal of the effect in *tel1*Δ, where cells fail to detect the shortness of their telomeres and replicate them inappropriately late (Fig. 1). The opposite nature of these phenotypes implies that Tel1 and Rif1 have opposing actions in the pathway that controls telomere replication timing, with Rif1 enforcing the late replication of long or normal length telomeres, while Tel1 signals early replication of telomeres that are shortened. Loss of Rif1 impacts replication timing of many genomic regions [48] with subtelomeric regions most strongly affected [4], probably because telomeres are the main genomic Rif1 binding locations [18].

To test the relationship of Tel1 and Rif1 in the telomere replication timing control, we examined a *rif1Δ tel1*Δ double mutant. Deleting *RIF1* somewhat relieves the short telomere phenotype of *tel1*Δ (Fig. 3A), presumably reflecting an effect of Rif1 on the backup mechanisms that recognize critically short telomeres in the absence of Tel1 [24]. We tested whether the *rif1Δ tel1*Δ strain replicates its telomeres early (as in *rif1*Δ) or late (as in *tel1*Δ). We found that in *rif1*Δ* tel1*Δ cells, both Y′ and telomere-proximal sequences replicate very early, similar to their replication time in a *rif1*Δ single mutant (Fig. 3B & C; replication times shown in Fig. S6). The *rif1*Δ mutation is therefore epistatic to *tel1*Δ in control of telomere replication—consistent with the idea that Tel1 counteracts Rif1-mediated delay to telomere replication timing.

### Rif1 is phosphorylated at Tel1 consensus sites in a mutant with short telomeres

**Figure 4:**
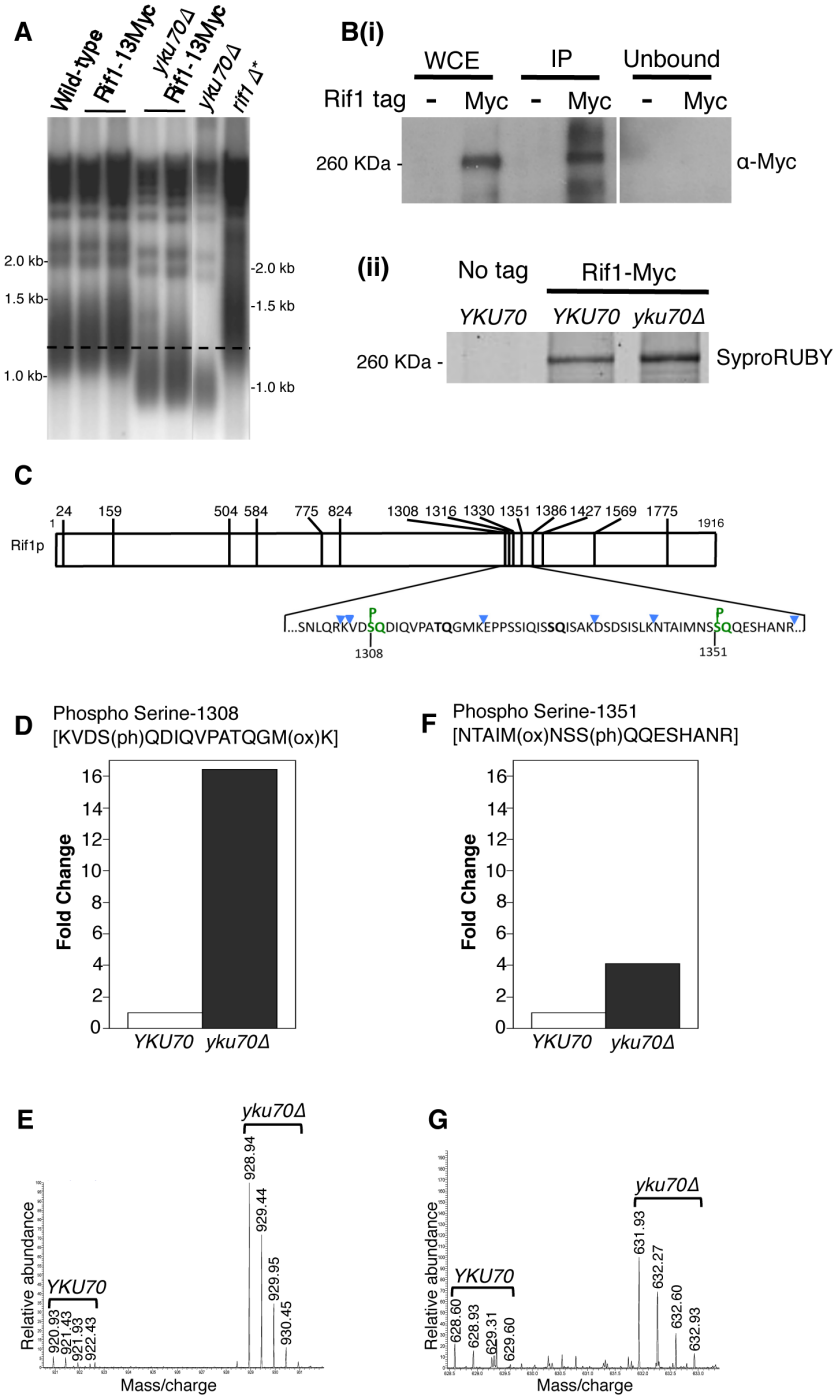
Rif1 is phosphorylated at Tel1 consensus sites in the short telomere mutant *yku70*Δ. (A) Telomere length gel confirms Myc-tagged Rif1 protein is functional. (B) Upper panel (i): Western blot analyzing Rif1-Myc protein in Whole Cell Extract (WCE), immunoprecipitated sample (IP) and supernatant (Unbound). All lanes show equivalent cell loading. Lower panel (ii): SyproRUBY-stained gel showing Rif1-Myc isolated from *YKU70* and *yku70*Δ strains, and mock IP from untagged control sample. Rif1 was quantified based on SyproRUBY gel bands and equivalent quantities mixed for SILAC mass spectrometry analysis. 260 kD marker position is indicated. The predicted size of Rif1-13Myc is 232 kD; Rif1-13Myc migration is slightly retarded relative to its predicted mass. (C) Cartoon of Rif1p sequence, illustrating the position of the 14 S/TQ sites. In enlarged sequence below S/TQ sequences are bold, and colored green are the two sites identified as phosphorylated in an initial mass spectrometry run (carried out using Rif1-Myc from *YKU70* and *yku70*Δ strains). Blue arrowheads indicate trypsin digestion sites. (D) Plot of SILAC ratio for phosphorylated peptide containing S-1308 in *yku70*Δ relative to *YKU70* (16.4× increased). In this and similar plots relative values are normalized during processing to the median H/L ratio of all Rif1 peptides. (E) MS spectrum showing raw results for the same S-1308 phosphorylated peptide [KVDS(ph)QDIQVPATQGM(ox)K], with light (R0K0) peptide from *YKU70* on left and heavy (R10K8) peptide from *yku70*Δ on right. (F) & (G) show equivalent SILAC analysis for the S-1351 phosphorylated peptide NTAIM(ox)NSS(ph)QQESHANR (4.1× increased in Δ*yku70* relative to *YKU70*). Strains (BY4741 strain background) are SHY201 (untagged wild-type), ASY25 (*YKU70 RIF1-13Myc*), ASY30 (*yku70*Δ *RIF1-13Myc*), Y00870 (untagged *yku70*Δ) and HYLS44 (*rif1*Δ*; asterisk indicating A364a strain background). An initial, non-SILAC, mass spectrometry analysis depicted in C used W303 Rad5+ strains YSM20 (*YKU70 RIF1-13Myc*) and ASY17 (*yku70*Δ* RIF1-13Myc*).

Since Tel1 is actively recruited to shortened telomeres, we hypothesized that Tel1 may act to prevent or ‘switch off’ the delaying effect of Rif1 on nearby replication origins. The Rif1 protein sequence contains multiple S/TQ motifs, corresponding to the consensus sequence for Tel1-mediated phosphorylation [27], [29], so Rif1 is a potential target for Tel1 kinase activity. We therefore tested by mass spectrometry whether Tel1 phosphorylates Rif1. Using Myc-tagged Rif1 that retains almost complete protein functionality (as assayed by telomere length, Fig. 4A), we devised an immunoprecipitation procedure to pull down the majority of cellular Rif1 (Fig. 4B). Initial high-resolution mass spectrometry identified multiple phosphorylated peptides in Rif1 from both *YKU70* and *yku70*Δ strains, including two phosphorylation sites corresponding to Tel1 consensus sequences, one at Serine-1308 (within the sequence…KVDSQDIQ…) and the other at Serine-1351 (…MNSSQQE…) (Fig. 4C). Rif1 S-1308 phosphorylation is not previously described; while S-1351 was identified as phosphorylated in response to DNA damage by MMS [49].

Identification of these phosphorylated SQ sites suggests that Rif1 may indeed be a target for Tel1 kinase, perhaps specifically at shortened telomeres which recruit Tel1. We used the comparative proteomic method of SILAC to compare phosphorylation levels in wild-type cells with the short telomere *yku70*Δ mutant. Phosphorylation at both sites were increased in the *yku70*Δ mutant, by about 16-fold at S-1308, and about 4-fold at S-1351 (Fig. 4D–G; Dataset S1). The corresponding unphosphorylated peptides were not increased in the *yku70*Δ strain (Fig. S7; Dataset S1). These results show that phosphorylation of these Rif1 SQ motifs is increased in the shortened telomere context of *yku70*Δ.

### Tel1 is required for phosphorylation of Rif1 Serine-1308

**Figure 5:**
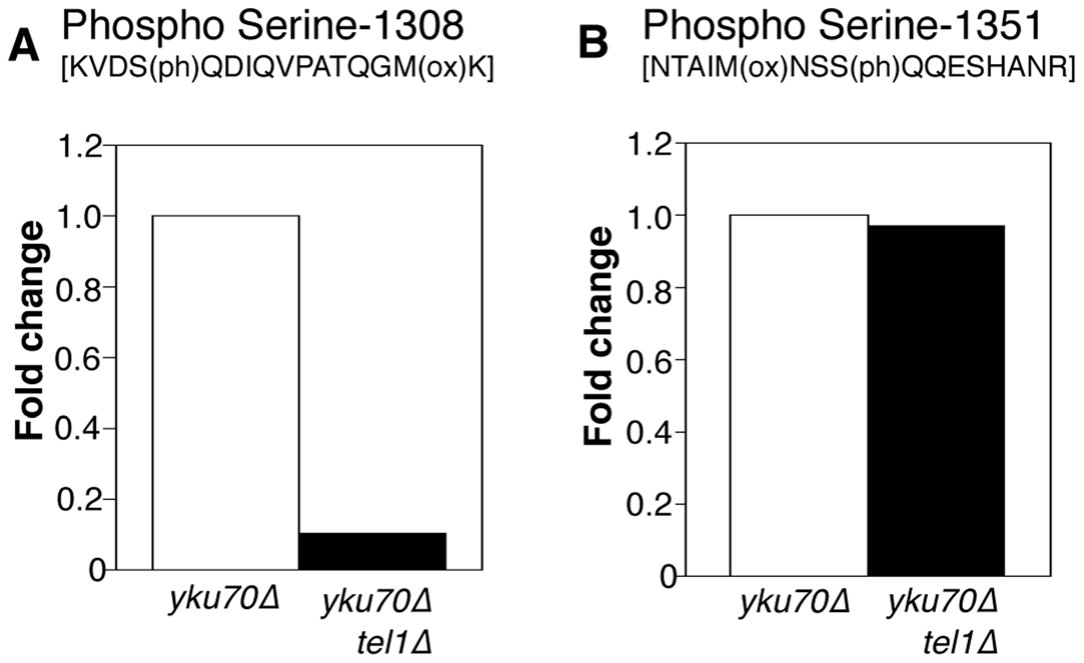
Phosphorylation of Rif1 Serine-1308 depends on Tel1. (A) Plots shows relative levels of the S-1308 phosphorylated peptide [KVDS(ph)QDIQVPATQGM(ox)K] in *yku70*Δ (Light-labeled R0K0) and *yku70*Δ* tel1*Δ (Heavy-labeled R10K8) strains. H/L ratio is 0.10. (B) Equivalent plot for S-1351 phosphorylated peptide NTAIM(ox)NSSQQESHANR. H/L ratio is 0.97048. Strains used are ASY30 (*yku70*Δ* RIF1-13Myc*), and ASY46 (*yku70*Δ* tel1*Δ *RIF1-13Myc*).

To address whether Tel1 kinase mediates phosphorylation of Rif1 at S-1308 and S-1351, we used a similar SILAC strategy to test whether the phosphorylation levels are decreased when Tel1 is unavailable. This experiment was carried out in the *yku70*Δ background where peptides containing phosphorylated S-1308 and S-1351 residues are reliably detected. Peptides from heavy-labeled *yku70*Δ* tel1*Δ cells were compared with those from light-labeled *yku70*Δ cells. The S-1308 phosphorylated peptide was abundant in the *yku70*Δ mutant, but was 10-fold reduced in the *yku70*Δ* tel1*Δ strain (Fig. 5A; Dataset S2). A longer peptide covering the same phosphorylated S-1308 residue was also greatly reduced in *yku70*Δ* tel1*Δ (Fig. S8A; Dataset S2), while its unphosphorylated equivalent showed no significant change (Fig. S8C; Dataset S2). In contrast, levels of the S-1351 phosphorylated peptide were largely unchanged in *yku70*Δ* tel1*Δ when compared to *yku70*Δ (Fig. 5B). Based on this analysis, we propose that Tel1 directly phosphorylates S-1308. However, we cannot exclude the possibility that Tel1 activates a different SQ-directed kinase that phosphorylates Rif1 S-1308 at short telomeres. If Tel1 contributes to phosphorylation at S-1351, its role can be substituted by a different kinase (probably Mec1 since [49] showed S-1351 phosphorylation requires one or other of Mec1 and Tel1). Alternatively, Mec1 may be solely responsible for S-1351 phosphorylation.

Fig. S9 provides a summary of the phosphorylation sites identified on Rif1 in these proteomic analyses. This study identified a cluster of phosphorylated DDK and CDK consensus sites close to the Rif1 N-terminus, which in a separate investigation were shown to regulate Protein Phosphatase 1 recruitment by Rif1 [21].

### Mutation of the Rif1 phosphorylated S/TQ cluster does not prevent the effect of telomere length on replication timing

**Figure 6:**
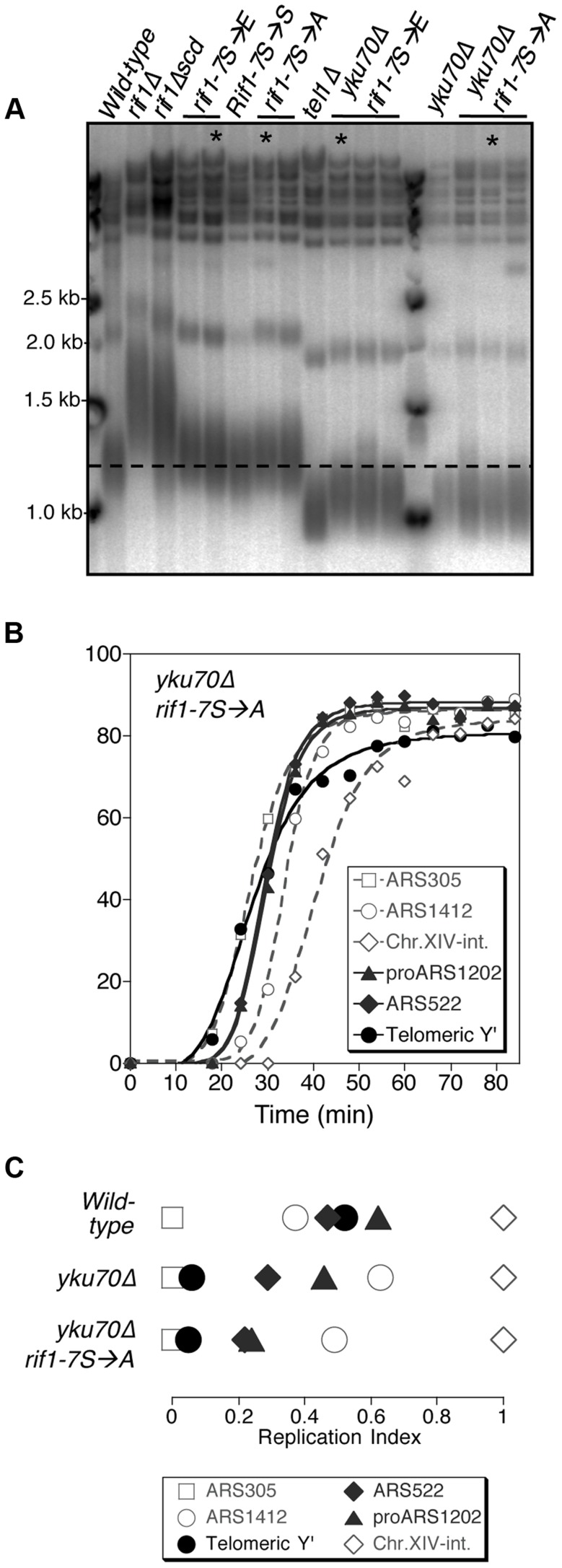
Non-phosphorylatable *rif1-7S*→*A* does not delay early replication of short *yku70*Δ telomeres. (A) Telomere length analysis of Rif1 phospho-site mutants. Genomic DNA was extracted from the indicated strains and telomere length analyses performed as described. Smear indicates average length of Y′ telomeres. *rif1*Δ*scd* represents an internal deletion within the *RIF1* C-terminal region made as a strain construction intermediate (see Supplementary Materials & Methods). *RIF1-7S*→*S* represents a *RIF1* reconstruction, where wild-type sequence was re-inserted into *rif1*Δ*scd* to control for telomere length recovery. (B) Replication program of *yku70*Δ *rif1-7S*→*A*, released from an α-factor block at 30°C. Sequences analyzed are as in Fig. 1. (C) Replication indices from *yku70*Δ* rif1-7S*→*A* experiment shown in B, along with values from wild-type and *yku70*Δ experiments from Fig. 1B&C. Strains in part A are BB14-3a (wild-type), HYLS44 (*rif1*Δ), ASY5 (*tel1*Δ), AW99 (*yku70*Δ), ASY51 (*rif1*Δ*scd*); ASY81 (*RIF1-7S→S*). For *rif1-7S*→*A* asterisk indicates ASY69, used for replication timing in Fig. S11; for *rif1-7S*→*E* asterisk indicates ASY73, used for replication timing in Fig. S12; for *yku70*Δ* rif1-7S*→*A* asterisk indicates ASY76, used for replication timing in Fig. 6 B&C and S10; for *yku70*Δ* rif1-7S*→*E* asterisk indicates ASY78.

Our results suggest a model in which Tel1-mediated phosphorylation of Rif1 antagonizes the delaying effect of Rif1 on telomeric and telomere-proximal replication origins at short telomeres. We constructed a Rif1 mutant where the relevant serine residues are replaced by alanine, to test whether this non-phosphorylatable construct constitutively delays replication, preventing early replication of short telomeres. We replaced the serine or threonine residue with alanine at all seven of the potential Tel1 phosphorylation sites (SQ and TQ motifs) between 1308 and 1569 in the Rif1 amino acid sequence, to construct a *rif1-7S→A* allele. We mutated the entire cluster of S/TQ motifs since it could contain phosphorylation sites not detected proteomically and because preventing phosphorylation of one of these residues might re-direct kinase activity to a nearby consensus site. Telomere length was hardly affected by this *rif1-7S→A* allele, or by an phospho-mimetic equivalent *rif1-7S→E* glutamate substitution allele, in either *YKU70* or *yku70*Δ backgrounds (Fig. 6A)—confirming that these substitutions do not ablate Rif1 protein function. We examined the replication program of the *rif1-7S→A* mutant in the short telomere (*yku70*Δ) background. We found that telomeres still replicate early (Fig. 6B), with Y′ elements replicating at a similar time to the early marker sequence ARS305 (Fig. 6C & S10), equivalent to the *yku70*Δ mutant. The non-phosphorylatable *RIF1* allele therefore does not prevent the early replication of short telomeres, implying that phosphorylation of the Rif1 S/TQ cluster is not essential for Tel1 to drive early replication of short telomeres. The *rif1-7S→A* mutation similarly caused minimal change to the replication timing program in a *YKU70* background (Fig. S11). We also tested the replication program of the *rif1-7S→E* allele designed to mimic a phosphorylated form of Rif1. In this *rif1-7S→E* mutant telomeres still replicate at approximately the same time as late origin ARS1412 (Fig. S12).

The phenotypic analyses of the *yku70*Δ* rif1-7S→A* and *rif1-7S→E* mutant therefore suggest that Tel1-mediated phosphorylation of the Rif1 S/TQ cluster is not necessary or sufficient to drive early replication. They do not however exclude the possibility that Tel1-mediated Rif1 S/TQ cluster phosphorylation could contribute to early replication of short telomeres. Indeed a very slight advancement (3–4 min; Fig. S12 B&C) in telomere replication time in the *rif1-7S→E* allele may be consistent with this idea. One possibility is that phosphorylation of Rif1 S-1308, S-1351 and nearby S/TQ sites is integrated with other, redundant mechanisms to ensure that shortened telomeres replicate early.

## Discussion

In investigating controls over telomere replication timing, we discovered that Tel1 specifies the early replication of short telomeres, as assessed either using a short telomere mutant (*yku70*Δ; Fig. 1) or by analyzing origin activation close to an induced short telomere (Fig. 2). Rif1 specifies late replication of normal telomeres, and epistatic analysis indicated that Tel1 counteracts the delaying effect of Rif1 on telomere replication time. Phosphoproteomic analysis of endogenous *S. cerevisiae* Rif1 revealed at least two SQ motifs to be phosphorylated. Phosphorylation at these sites is increased in a short telomere mutant, with phosphorylation at Serine-1308 completely dependent on the presence of Tel1. However, corresponding Rif1 alanine substitution mutants did not prevent early replication of telomeres in a *yku70*Δ background, indicating that phosphorylation of Rif1 by Tel1 at S-1308, S-1351, or nearby consensus sites within the Rif1 S/TQ cluster domain, cannot be the sole mechanism by which Tel1 drives early replication at short telomeres. While Rif1 phosphorylation could potentially contribute, Tel1 must mediate early replication of short telomeres through additional, possibly redundant, pathways.

**Figure 7:**
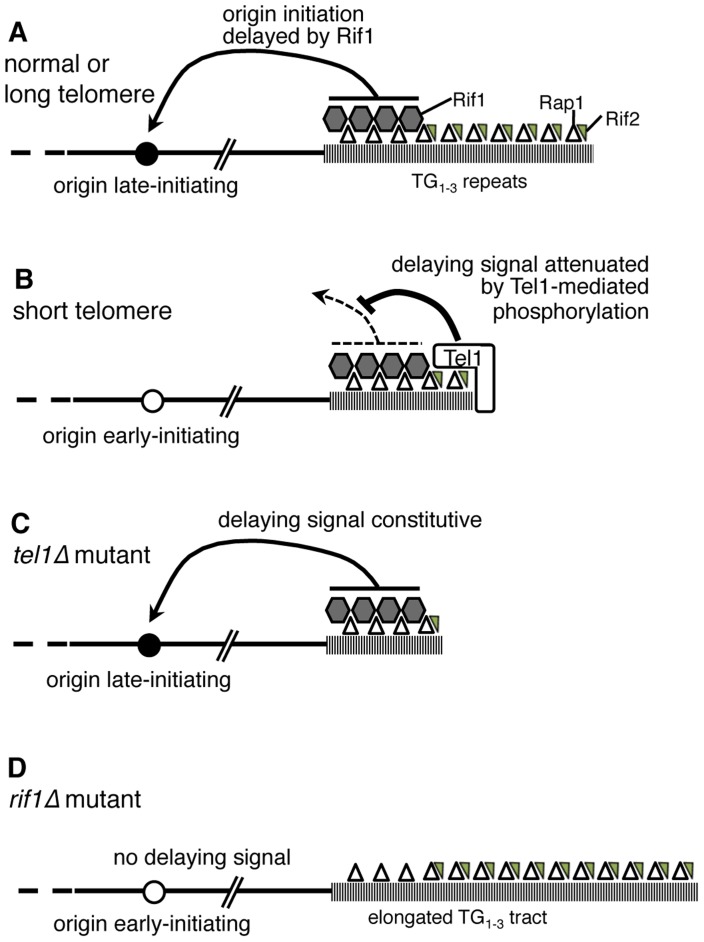
Model of replication timing control by Tel1 and Rif1. (A) In wild-type cells, terminal TG_1–3_ tract is bound by Rap1 (open triangles) which recruits Rif1 (grey hexagons) and Rif2 (small grey triangles). If the telomere is normal in length, Rif1 signals to nearby origins (such as telomere-proximal Y′ or ARS522 origins) specifying their late replication time (filled circle). (B) If telomeres are short (as in a *yku70*Δ mutant) Tel1 kinase is recruited and neutralizes the Rif1 delaying signal, so that nearby origins initiate early (white circle). (C) In *tel1*Δ mutant cells, the delaying effect of Rif1 cannot be neutralized so that nearby origins initiate late despite the short telomeres. (D) A *rif1*Δ mutant lacks the delaying signal, with the result that nearby origins initiate replication early despite their extended TG_1–3_ repeat length.

*S. pombe*, *S. cerevisiae* and human Rif1 proteins all negatively regulate DNA replication genome-wide [18]–[21], and very recently it was shown that Rif1 recruits Protein Phosphatase 1 to control DNA replication [21]–[23]. The stimulatory effect of removing *S. cerevisiae* Rif1 on the overall replication program is reflected by a shortened S phase (Fig. 3B & S6A: S phase duration is 21.5 min in wild-type but 15.3 min in *rif1*Δ). Within the generally shortened S phase of the *rif1*Δ mutant telomeres are more dramatically affected, with telomere-associated sequences shifting their replication time from the latter half to the early part of S phase (Fig. 3C). Proximity of Rif1 binding sites has been suggested to determine the susceptibility of replication origin initiation to Rif1-mediated repression [18], and the delaying effect of Rif1 on replication is probably focused at chromosome ends by the preferential association of Rif1 with telomeres, as illustrated in Fig. 7A, explaining why telomere regions show the largest shift in replication timing when Rif1 is removed (Fig. 3C). It is possible that non-telomeric Rif1 also contributes to the late replication of telomere regions.

Removing both Tel1 and Rif1 leads to a phenotype that is essentially equivalent to a *rif1*Δ single mutant—that is, in the absence of Rif1, it becomes largely irrelevant for telomere replication timing whether Tel1 is present (Fig. 3C). For this reason, our results support a model where Tel1 affects replication timing by counteracting the delaying action of Rif1 on telomere replication, as illustrated in Fig. 7A & B. If non-telomeric Rif1 contributes to late replication of subtelomeric regions, its effect is presumably also neutralized by Tel1.

 We envisage two modes through which Tel1 could counteract the delaying effect of Rif1 on origin initiation. First, phosphorylation of Rif1 by Tel1 at SQ sites might ‘switch off’ the Rif1 repressive effect. We identified Rif1 as a target of Tel1 phosphorylation at shortened telomeres, but mutating the sites identified, along with neighboring potential phosphorylation sites, did not dramatically impact telomere replication timing. This observation argues that Rif1 phosphorylation cannot be solely responsible for Tel1-driven early telomere replication, while leaving open the possibility that Rif1 phosphorylation acts redundantly with other control mechanisms. It is possible that Rif1 contains additional functionally critical Tel1 phosphorylation sites not identified by our proteomic analysis. It is also conceivable that phosphorylation of Rif1 by Tel1 at non-consensus (i.e. non-S/TQ) sites might contribute to replication timing control. A previous study [28] showed that a Dun1 substrate lacking any SQ consensus was still phosphorylated by Tel1 kinase, and noted that ATM (the mammalian homolog of Tel1) phosphorylates non-canonical sites in the tumor suppressor BRCA1 [50]. Intriguingly, in the *yku70*Δ mutant we observed a 2 to 4-fold increase in phosphorylation levels of five serine or threonine residues that are not followed by glutamine (Rif1 S-1338, S-1355, S-1362, T-1367, and S-1694; Fig. S9 & Dataset S1).

Second, Tel1 could prevent the Rif1-mediated replication delay by phosphorylating a different telomeric protein. A number of telomeric proteins have been identified as likely or possible targets of Tel1 phosphorylation, including Cdc13 [29], Xrs2, and Mre11 [28]. While they cannot be formally excluded, none of these proteins is directly implicated in controlling replication origin activation. It seems more likely that Tel1 counteracts the Rif1-mediated delay by phosphorylating an unidentified component of the molecular pathway through which Rif1 restrains origin activation. Such a mechanism could act redundantly with Tel1-mediated Rif1 phosphorylation to neutralize the Rif1 replication-delaying signal. Tel1 appears to have multiple targets at telomeres [28], [29], which may act in concert to produce biological function, so that ablating any particular phosphorylation event has rather mild effects.

A third possibility is that telomere replication timing control depends on multiple mechanisms some of which do not involve Rif1, although the strong effect of Rif1 loss on telomere replication (Fig. 3) does suggest it is the most central controller of telomere replication time. H2A-S129 phosphorylation depends on Tel1 in telomere-proximal regions [51], and a non-phosphorylatable (H2A-S129A) allele caused a slight delay to telomere replication in a *yku70*Δ background (unpublished observations); however, H2A-S129 phosphorylation is not elevated at shortened telomeres [51], inconsistent with H2A-S129 phosphorylation being a critical mediator of the early replication of short telomeres.

Phosphorylation of Rif1 may contribute to other telomeric functions. One possibility is that Tel1-mediated Rif1 phosphorylation counteracts repression of telomerase recruitment, favoring TG_1–3_ tract extension. Telomere length is not greatly altered by the *rif1-7S→A* or *rif1-7S→E* mutants (Fig. 6A)—although very slight telomere lengthening in some *rif1-7S→E* isolates hints that Rif1 phosphorylation might contribute to telomerase recruitment. As with replication timing, Rif1 phosphorylation may be one of a series of redundant mechanisms through which Tel1 regulates telomerase recruitment—another potential pathway being phosphorylation of Cdc13 [16]. A further role for Rif1 phosphorylation might involve regulation of anti-checkpoint function at telomeric DNA damage sites [43], [52].

To summarize, we have identified an important new function for Tel1—namely, driving the early replication of shortened telomeres. Our results suggest that Tel1 exerts this function by neutralizing the delaying effect of telomeric Rif1 on nearby replication origins. Tel1 also directs phosphorylation of Rif1, which may contribute to replication timing control along with other mechanisms that impact on origin initiation time. Since Rif1 and Tel1 are conserved and play similar roles in replication timing control and coordination of DNA repair in higher eukaryotes as in yeast, our discoveries are likely to illuminate general functions of these proteins.

## Materials and Methods

### Yeast strains

Yeast strains are listed in Supplemental Table S1. Gene knockouts and tagging used standard PCR-based insertion methods, confirmed by PCR analysis; see Supplemental material (Text S1) for details of specific strain constructions. Primer sequences are available on request.

### Analysis of replication time

The replication time of specific sequences was measured using the dense isotope transfer procedure [53], [54] in cells released from α-factor at 30°C, probing for genomic EcoRI fragments as described previously [8].

### Two-dimensional gel analysis of replication intermediates

Inducible HO cut strains were initially grown in YP medium containing 2% raffinose with 0.01% glucose (to allow adaptation to raffinose), and then grown for 24–48 hours in 2% raffinose at 30°C before blocking with 200 nM α-factor. Then galactose was added to obtain a final concentration of 4%, to induce HO endonuclease. After 5.5–6 hr, the cells were then released by the addition of pronase with simultaneous addition of 200 mM hydroxyurea, and harvested 2 h later. DNA was prepared using the NIB-n-grab method [55] digested with HindIII followed by 2-dimensional gel electrophoresis under standard conditions [47]. HO cutting efficiency was confirmed by Southern blot analysis of XmnI-digested DNA.

### Immunoprecipitation of Rif1-Myc

Immunoprecipitation of Rif1 was carried out as described [56] with modifications as described in Supplementary Material (Text S1). Protein concentrations were estimated using the RCDC kit (Bio-rad).

### Western blotting and SyproRUBY staining

Immunoprecipitated proteins were eluted in 1× SDS sample buffer (Invitrogen) with 5% 2-Mercaptoethanol. Cellular equivalent protein samples were separated by SDS PAGE (Novex 8–16% Tris-Glycine gels, Precast; Invitrogen) and wet blotted using 1× Towbin buffer with 10% Methanol onto PVDF membrane (Hybond-P, GE Healthcare). Rabbit anti-Myc (ab9106, Abcam) was used to detect epitope-tagged *RIF1*, with secondary antibody AP-conjugated anti-Rabbit IgG (S3731, Promega). Detection substrate was CDP-Star (Perkin Elmer) using Medical X-ray (Fuji) film. For quantification of amount of Rif1 protein, a similar gel was stained overnight using SyproRUBY total protein staining solution (Bio-rad) and quantified with a Fuji Phosphorimager (FLA3000) at 473 nm with O580 filter and FujiFILM ImageGauge (software V4.21).

### SILAC sample preparation and mass spectrometry analysis

SILAC samples were prepared based on the procedure described [57]. To compare *yku70*Δ with wild-type (Fig. 4), yeast strain AYS30 was labeled with heavy L-ARGININE:HCL (U-13C6: U-15N4; CNLM-539-H; Cambridge Isotope Laboratory) and L-LYSINE:2HCL (U-13C6; U-15N2, CNLM-291-H; Cambridge Isotope Laboratory) [R10K8] and ASY25 was labeled with light alternatives [R0K0] for at least ten generations. To compare *yku70*Δ* tel1*Δ with *yku70*Δ (Fig. 5), ASY46 was labeled with heavy Lysine and Arginine [R10K8] and ASY30 was labeled with light alternatives [R0K0] for at least ten generations, and subjected to immunoprecipitation as described above. Immunoprecipitated Rif1 was quantified by SYPRORuby staining. Equal masses of Rif1 were then mixed and run on a Novex 8–16% Tris-Glycine gel, and the Rif1 band was excised for analysis by high-resolution mass spectrometry (FingerPrints Proteomics, University of Dundee) as described in Supplementary Information (Text S1).

### Telomere length analysis

Genomic DNA was digested with XhoI, separated on a 1.5% agarose gel and transferred to neutral membrane (MP Biomedicals) by Southern blotting. Terminal restriction fragments were detected using a probe directed against the TG repeats.

## Supporting Information

Dataset S1 List of peptides identified in the SILAC analysis of wild-type (*YKU70*) versus *yku70*Δ with H/L ratios and peptide identification details. First worksheet explains each column in subsequent sheets; second worksheet lists the most significant identified phospho-peptides; third worksheet lists all Rif1 peptides identified, both modified and unmodified, with corresponding evidence basis.
(XLS)

Dataset S2 List of peptides identified in the SILAC analysis of *yku70*Δ versus *yku70*Δ* tel1*Δ with H/L ratios and peptide identification details. First worksheet explains each column in subsequent sheets; second worksheet lists the most significant identified phospho-peptides; third worksheet lists all Rif1 peptides identified, both modified and unmodified, with corresponding evidence basis.
(XLS)

Figure S1 Replication times show Tel1 is required for early replication of short *yku70*Δ telomeres. Replication times (from experiments in Fig. 1B) plotted relative to the replication time of early origin ARS305 (set to time = 0 min). Strains are BB14-3a (wild-type), ASY5 (*tel1*Δ), AW99 (*yku70*Δ) and ASY13 (*yku70*Δ* tel1*Δ; corresponding to second isolate in part A); all are in A364a background as listed in Table S1.
(PDF)

Figure S2 Strain-dependent effects of *yku70*Δ mutation in A364a and BY4741 backgrounds. Telomere length analysis shows that in A364a background, telomeres in a *yku70*Δ mutant are longer than in a *tel1*Δ mutant. In the BY4741 strain background, *yku70*Δ and *tel1*Δ have similarly very short telomeres. Strain-dependence of the effect of the *yku70*Δ mutation on telomere length has been observed previously (compare references [36], [37] and [38]–[40] in main reference list). Strains used in the A364a strain background are BB14-3a (wild-type), ASY5 (*tel1*Δ), AW99 (*yku70*Δ) and ASY13 (*yku70*Δ* tel1*Δ); and in the BY4741 strain background are Y0000 (wild-type), Y03114 (*tel1*Δ) and Y00870 (*yku70*Δ).
(PDF)

Figure S3 Confirmation that Tel1 is required for early replication of short telomeres. (A) Replication kinetics of various genomic sequences in wild-type and short telomere mutants *yku70*Δ, *tel1*Δ and *yku70*Δ* tel1*Δ. Plots and symbols as in Fig. 1B, in these repeats of experiments in Fig. 1 & S1. (B) Replication indices from experiments in A. (C) Replication times from experiments in A, plotted relative to the replication time of early origin ARS305 (set to time = 0 min). Strains are BB14-3a (wild-type), ASY5 (*tel1*Δ), AW99 (*yku70*Δ) and ASY13 (*yku70*Δ* tel1*Δ; corresponding to second isolate in part A); all are in A364a background as listed in Table S1.
(PDF)

Figure S4 Tel1 is required for efficient activation in hydroxyurea of the ARS700.5 origin neighboring an induced short telomere. (A) Evaluating the efficiency of HO cutting used to generate a single short telomere. Cartoon of inducible short telomere construct is shown in main Fig. 2A. Cells were grown in 2% Raffinose (Asynchronous), arrested with α factor in 4% Galactose (lanes 2–4 and 10–12) and released into S phase in the presence of HU (lanes 5–8 and 13–16). XmnI-digested DNA samples were probed for the 5′ part of *ADE2* (see Fig. 2A). Percentage cutting is indicated, prior to release and 120 min after release into HU. Asterisk indicates ARS1412 fragment, also probed in this experiment. (B) Quantification of the bubble arc in ARS700.5 relative to loading as assessed by the intensity of the ‘1N spot’ of non-replicating DNA. Boxes illustrate the area used for intensity quantification. (C) Table showing relative intensity values of the bubble arc and 1N spots for ARS700.5, ARS305 and ARS1412. Bubble arc values were extracted from long 2D gel exposures and 1N spot values extracted from short 2D gel exposures, to maintain phosphorimager signal linearity. After normalization for loading, the reduction in origin activation of ARS700.5 was 4.8-fold in the *tel1*Δ strain relative to *TEL1*. Origin activation levels were in contrast hardly affected for early origin ARS305 and late origin ARS1412. Gels used for quantification shown in Fig. 2C. Strains used are SMKY10 (*TEL1*) and SMKY13 (*tel1*Δ).
(PDF)

Figure S5 Activation of ARS700.5 depends on the length of nearby telomeric repeats. 2D gel analysis of replication intermediates present at ARS700.5 in strains with either long (TG250) or short (TG80) telomeric TG repeats adjacent to the HO cut site. Strains used are YAB1356 (TG250) and SMKY10 (TG80).
(PDF)

Figure S6 Telomeres replicate early in a *rif1*Δ mutant. (A) Replication times (from experiments in Fig. 3B), plotted relative to the replication time of early origin ARS305 (set to time = 0 min), along with values from wild-type and *tel1*Δ experiments from Fig. 1 and S1). Strains are HYLS44 (*rif1*Δ) and ASY14 (*rif1*Δ* tel1*Δ; corresponding to first isolate in Fig. 3A). (B) 2D gel analysis of replication intermediates present at ARS700.5 in *RIF1* (left) and *rif1*Δ (right) strains following short telomere induction with HO endonuclease. Cells were analyzed following release into HU as described for Fig. 2. Strains are SMKY10 (*RIF1*) and SMKY15 (*rif1*Δ).
(PDF)

Figure S7 Abundance of non-phosphorylated Rif1 peptides is not increased in *yku70*Δ. (A) MS spectrum showing non-phosphorylated peptide KVDSQDIQVPATQGM(ox)K, with light (unlabeled) peptide from wild-type (R0K0) and heavy-labeled peptide from *yku70*Δ (R10K8). This peptide represents the unphosphorylated form of the S-1308 phosphorylated peptide shown in Fig. 4E. (B) MS spectrum showing the non-phosphorylated peptide NTAIM(ox)NSSQQESHANR, with light (unlabeled) peptide from wild-type (R0K0) and heavy-labeled peptide from *yku70*Δ (R10K8). This peptide represents the unphosphorylated form of the S-1351 phosphorylated peptide shown in Fig. 4G.
(PDF)

Figure S8 Abundance of a longer Rif1 peptide, phosphorylated at Serine-1308, is decreased in the absence of Tel1. (A) Plots shows relative levels of S-1308 phosphorylated peptide [KVDS(ph)QDIQVPATQGM(ox)KEPPSSIQISSQISAK] in *yku70*Δ (Light-labeled) and *yku70*Δ* tel1*Δ (Heavy-labeled) strains. This is a longer peptide encompassing the same sequence as the peptide in Fig. 5A, containing a lysine not cleaved during the trypsin digestion. (B) MS spectrum of the same peptide [KVDS(ph)QDIQVPATQGM(ox)KEPPSSIQISSQISAK] comparing relative abundance in *yku70*Δ (R0K0-labeled) and *yku70*Δ*tel1*Δ (R10K8-labeled). (C) MS spectrum comparing abundance of the non-phosphorylated form of the peptide KVDSQDIQVPATQGM(ox)KEPPSSIQISSQISAK in *yku70*Δ (R0K0-labeled) and *yku70*Δ* tel1*Δ (R10K8-labeled).
(PDF)

Figure S9 Summary of phosphorylation sites identified in Rif1. Rif1 amino acid sequence with phosphorylation sites identified and changes observed in the experiments shown in Figures 4 and 5. Potential Tel1/Mec1 phosphorylation consensus (S/TQ) sequences are underlined, while green bars above indicate PP1 interaction motifs. Identified phosphorylation sites with probability>0.7 are shown in red. ‘Linked’ phosphorylation sites (identified only on di- or tri- phosphorylated peptides) with probability>0.7 are shown in blue. Arrows represent fold change observed in phosphorylated peptides in SILAC experiments indicated. In most cases, there were comparable fold-changes where peptides were identified in mono- and di-phosphorylated forms. An exception was the di-phosphorylated peptide LHNGNIFT(ph)S(ph)PYK (indicated with blue asterisk), where the di-phosphorylated form was 10-fold increased in *yku70*Δ* tel1*Δ, relative to *yku70*Δ single mutant. A third Mec1/Tel1 phosphorylation consensus sequence was assigned as phosphorylated at Threonine-1569, but close inspection of the fragmentation profile revealed ambiguity of the assignment between S-1567 and T-1569. No arrows shown where fold change was <1.8×.
(PDF)

Figure S10 Replication times show that the non-phosphorylatable Rif1 does not delay the early replication of *yku70*Δ short telomeres. Replication times (from experiments in Fig. 6B), plotted relative to the replication time of early origin ARS305 (set to time = 0 min), along with values from wild-type and *yku70*Δ experiments from Fig. 1 and S1). Strains used are ASY76 (*rif1-7S→A yku70*Δ), BB14-3a (wild-type) and AW99 (*yku70*Δ).
(PDF)

Figure S11 Non-phosphorylatable Rif1 does not affect telomeric replication times in *YKU70* strain background. (A) Replication program of *rif1-7S*→*A*, released from an α-factor block at 30°C. Sequences analyzed are as in Fig. 1. (B) Replication indices from *rif1-7S*→*A* experiment shown in A, along with values from wild-type experiment from Fig. 1B&C. (C) Replication times (from experiments in A) plotted relative to the replication time of early origin ARS305 (set to time = 0 min). Strains used are ASY69 (*rif1-7S→A*) and BB14-3a (wild-type).
(PDF)

Figure S12 In the *rif1-7S→E* mutant telomere replication time is not advanced relative to ARS1412. (A) Replication program of *rif1-7S*→*E*, released from an α-factor block at 30°C. Sequences analyzed are as in Fig. 1. (B) Replication indices from *rif1-7S*→*E* experiment shown in A, along with values from wild-type experiment from Fig. 1B&C. (C) Replication times (from experiments in A) plotted relative to the replication time of early origin ARS305 (set to time = 0 min). Strains used are ASY73 (*rif1-7S→E*) and BB14-3a (wild-type).
(PDF)

Table S1 Yeast strains. Yeast strains used in this study are listed along with their source and the figures where used.
(DOC)

Text S1 Supplementary experimental procedures. Text file with in-detail procedures for strain construction, Rif1 immunoprecipitation, and mass spectrometry and data analyses.
(DOC)
