# Supplementary figures and images for: At Short Telomeres Tel1 Directs Early Replication and Phosphorylates Rif1
Source: PLoS Genet. 2014 Oct 16;10(10):e1004691. doi: 10.1371/journal.pgen.1004691 (PMC4199499; doi:10.1371/journal.pgen.1004691)

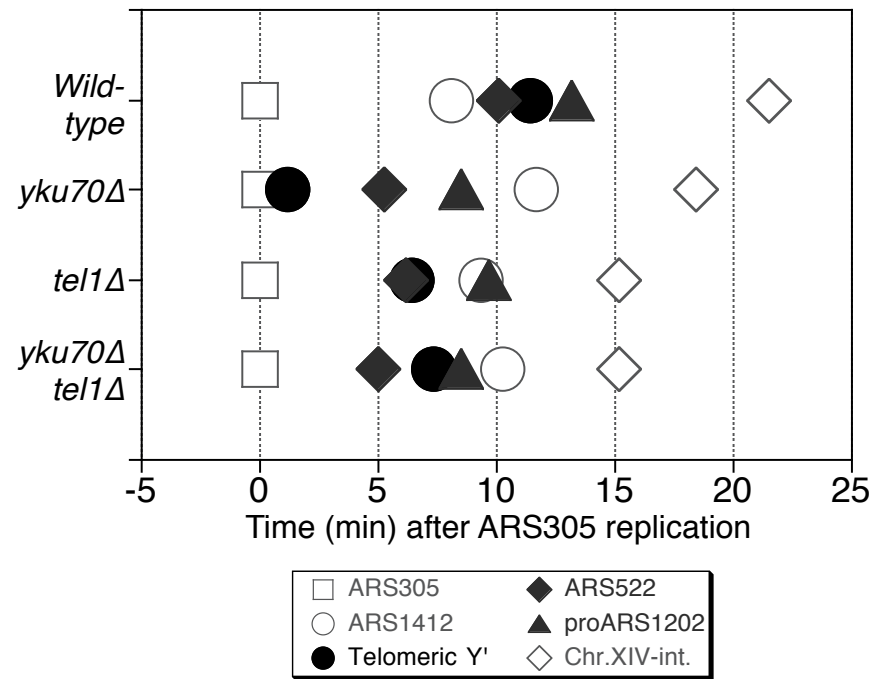

**Figure S1. Replication times show Tel1 is required for early replication of short *yku70*Δ telomeres.**

Supplement: Figure S1 — Replication times show Tel1 is required for early replication of short yku70Δ telomeres. Replication times (from experiments in Fig. 1B) plotted relative to the replication time of early origin ARS305 (set to time = 0 min). Strains are BB14-3a (wild-type), ASY5 (tel1Δ), AW99 (yku70Δ) and ASY13 (yku70Δ tel1Δ; corresponding to second isolate in part A); all are in A364a background as listed in Table S1. (PDF) [file pgen.1004691.s003.pdf]

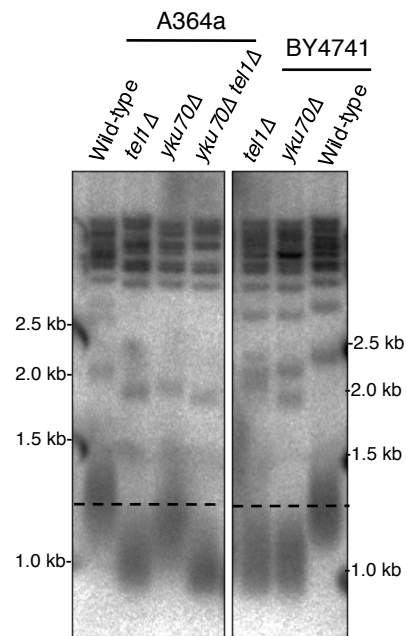

**Figure S2. Strain-dependent effects of *yku70Δ* mutation in A364a and BY4741 backgrounds.**

Supplement: Figure S2 — Strain-dependent effects of yku70Δ mutation in A364a and BY4741 backgrounds. Telomere length analysis shows that in A364a background, telomeres in a yku70Δ mutant are longer than in a tel1Δ mutant. In the BY4741 strain background, yku70Δ and tel1Δ have similarly very short telomeres. Strain-dependence of the effect of the yku70Δ mutation on telomere length has been observed previously (compare references [36], [37] and [38]–[40] in main reference list). Strains used in the A364a strain background are BB14-3a (wild-type), ASY5 (tel1Δ), AW99 (yku70Δ) and ASY13 (yku70Δ tel1Δ); and in the BY4741 strain background are Y0000 (wild-type), Y03114 (tel1Δ) and Y00870 (yku70Δ). (PDF) [file pgen.1004691.s004.pdf]

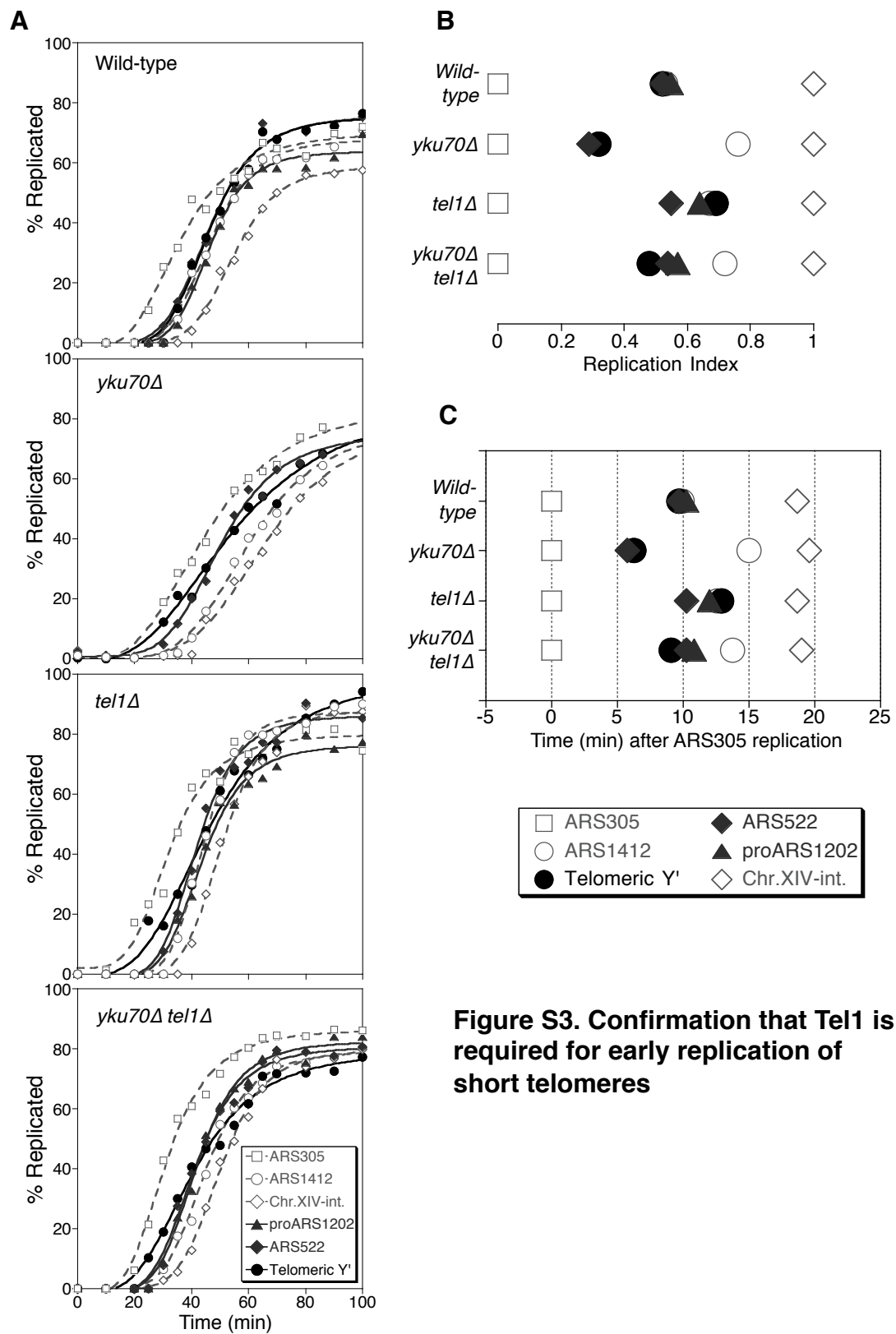

**Figure S3. Confirmation that Tel1 is required for early replication of short telomeres**

Supplement: Figure S3 — Confirmation that Tel1 is required for early replication of short telomeres. (A) Replication kinetics of various genomic sequences in wild-type and short telomere mutants yku70Δ, tel1Δ and yku70Δ tel1Δ. Plots and symbols as in Fig. 1B, in these repeats of experiments in Fig. 1 & S1. (B) Replication indices from experiments in A. (C) Replication times from experiments in A, plotted relative to the replication time of early origin ARS305 (set to time = 0 min). Strains are BB14-3a (wild-type), ASY5 (tel1Δ), AW99 (yku70Δ) and ASY13 (yku70Δ tel1Δ; corresponding to second isolate in part A); all are in A364a background as listed in Table S1. (PDF) [file pgen.1004691.s005.pdf]

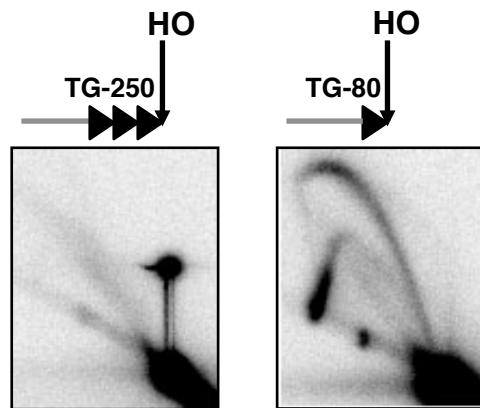

**Figure S5. Activation of ARS700.5 depends on the length of nearby telomeric repeats.**

Supplement: Figure S5 — Activation of ARS700.5 depends on the length of nearby telomeric repeats. 2D gel analysis of replication intermediates present at ARS700.5 in strains with either long (TG250) or short (TG80) telomeric TG repeats adjacent to the HO cut site. Strains used are YAB1356 (TG250) and SMKY10 (TG80). (PDF) [file pgen.1004691.s007.pdf]

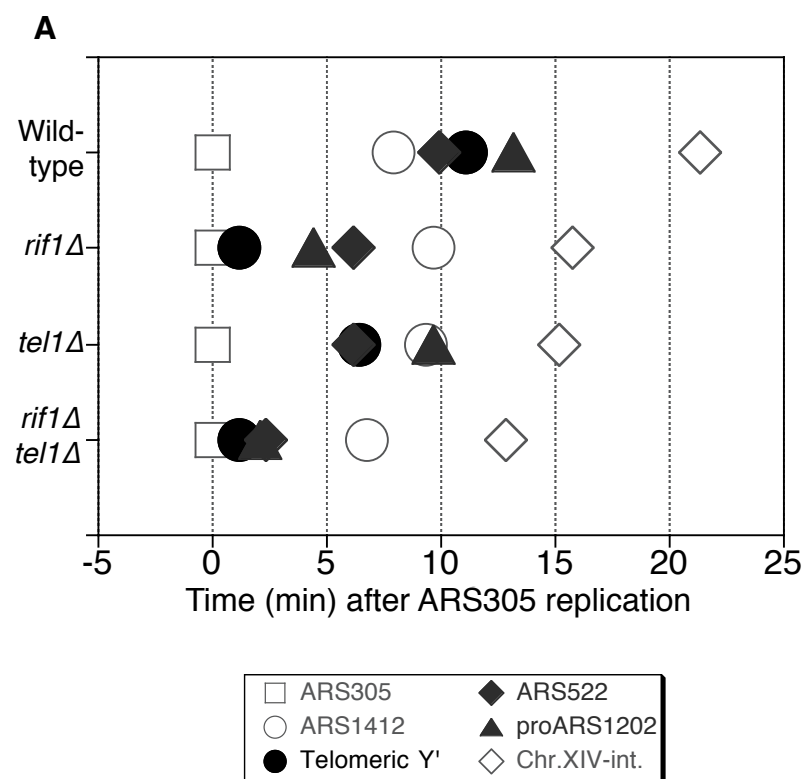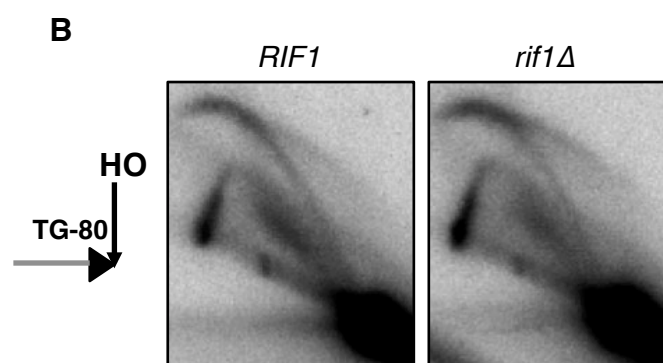

**Figure S6. Telomeres replicate early in a *rif1*Δ mutant.**

Supplement: Figure S6 — Telomeres replicate early in a rif1Δ mutant. (A) Replication times (from experiments in Fig. 3B), plotted relative to the replication time of early origin ARS305 (set to time = 0 min), along with values from wild-type and tel1Δ experiments from Fig. 1 and S1). Strains are HYLS44 (rif1Δ) and ASY14 (rif1Δ tel1Δ; corresponding to first isolate in Fig. 3A). (B) 2D gel analysis of replication intermediates present at ARS700.5 in RIF1 (left) and rif1Δ (right) strains following short telomere induction with HO endonuclease. Cells were analyzed following release into HU as described for Fig. 2. Strains are SMKY10 (RIF1) and SMKY15 (rif1Δ). (PDF) [file pgen.1004691.s008.pdf]

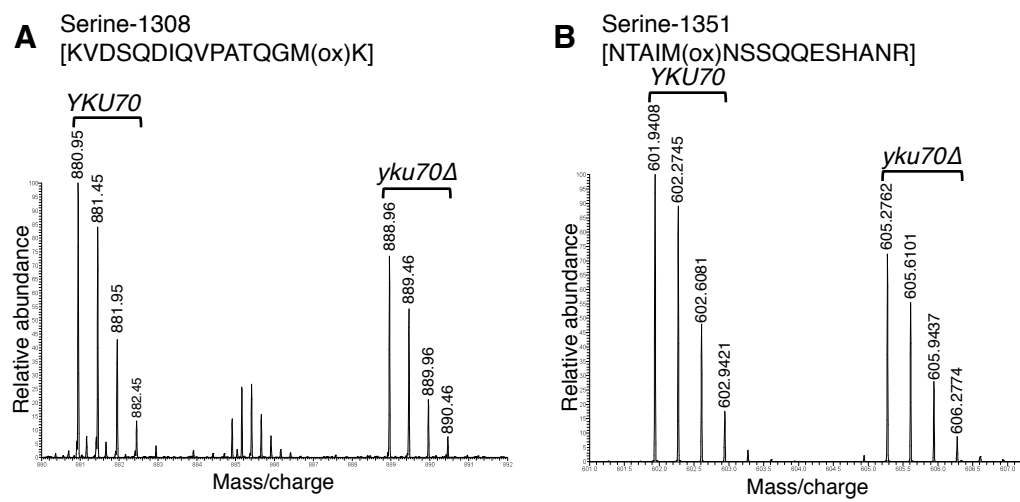

**Figure S7. Abundance of non-phosphorylated Rif1 peptides is not increased in *yku70Δ***

Supplement: Figure S7 — Abundance of non-phosphorylated Rif1 peptides is not increased in yku70Δ. (A) MS spectrum showing non-phosphorylated peptide KVDSQDIQVPATQGM(ox)K, with light (unlabeled) peptide from wild-type (R0K0) and heavy-labeled peptide from yku70Δ (R10K8). This peptide represents the unphosphorylated form of the S-1308 phosphorylated peptide shown in Fig. 4E. (B) MS spectrum showing the non-phosphorylated peptide NTAIM(ox)NSSQQESHANR, with light (unlabeled) peptide from wild-type (R0K0) and heavy-labeled peptide from yku70Δ (R10K8). This peptide represents the unphosphorylated form of the S-1351 phosphorylated peptide shown in Fig. 4G. (PDF) [file pgen.1004691.s009.pdf]

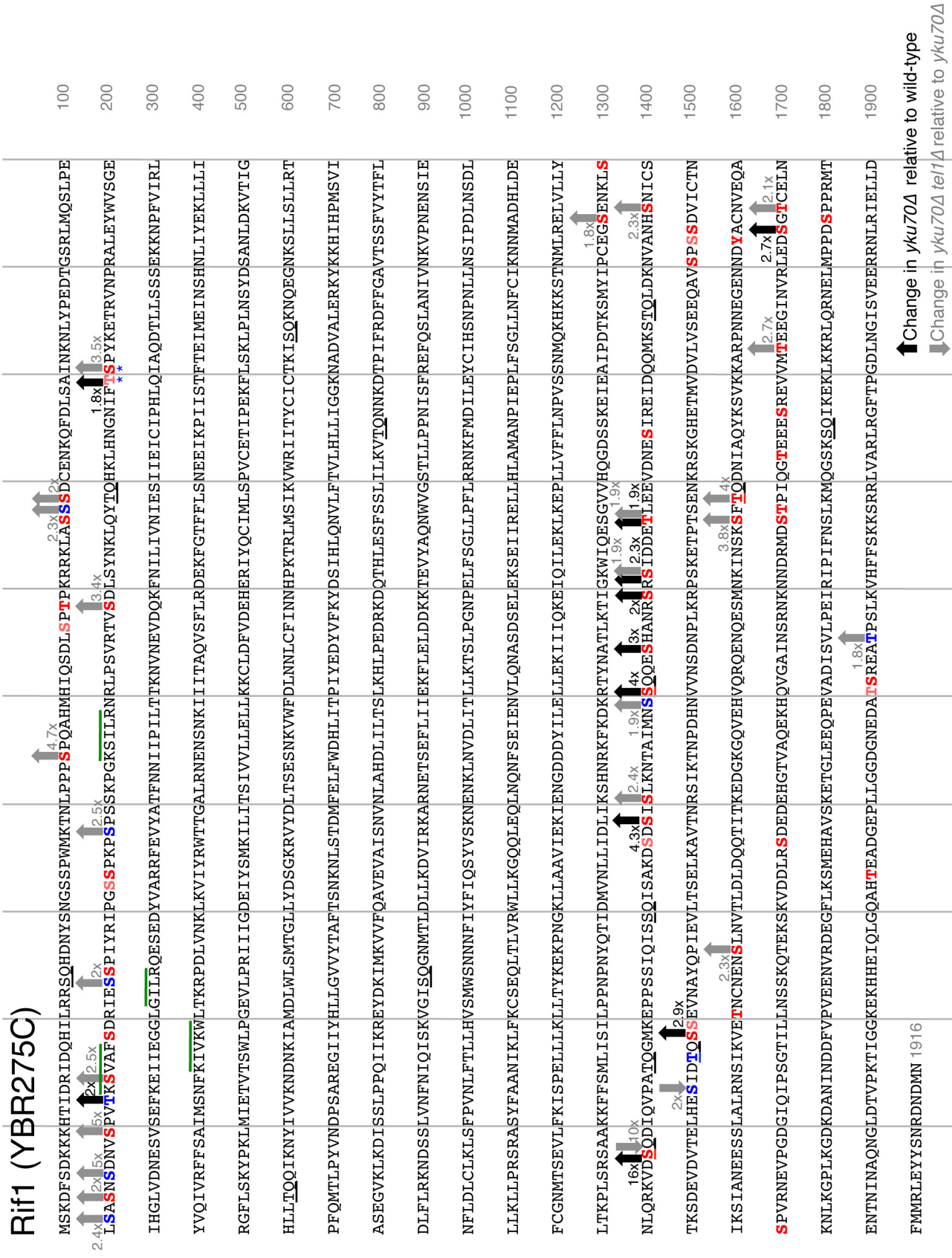

Figure S9. Summary of phosphorylation sites identified in Rif1

Supplement: Figure S9 — Summary of phosphorylation sites identified in Rif1. Rif1 amino acid sequence with phosphorylation sites identified and changes observed in the experiments shown in Figures 4 and 5. Potential Tel1/Mec1 phosphorylation consensus (S/TQ) sequences are underlined, while green bars above indicate PP1 interaction motifs. Identified phosphorylation sites with probability>0.7 are shown in red. ‘Linked’ phosphorylation sites (identified only on di- or tri- phosphorylated peptides) with probability>0.7 are shown in blue. Arrows represent fold change observed in phosphorylated peptides in SILAC experiments indicated. In most cases, there were comparable fold-changes where peptides were identified in mono- and di-phosphorylated forms. An exception was the di-phosphorylated peptide LHNGNIFT(ph)S(ph)PYK (indicated with blue asterisk), where the di-phosphorylated form was 10-fold increased in yku70Δ tel1Δ, relative to yku70Δ single mutant. A third Mec1/Tel1 phosphorylation consensus sequence was assigned as phosphorylated at Threonine-1569, but close inspection of the fragmentation profile revealed ambiguity of the assignment between S-1567 and T-1569. No arrows shown where fold change was <1.8×. (PDF) [file pgen.1004691.s011.pdf]

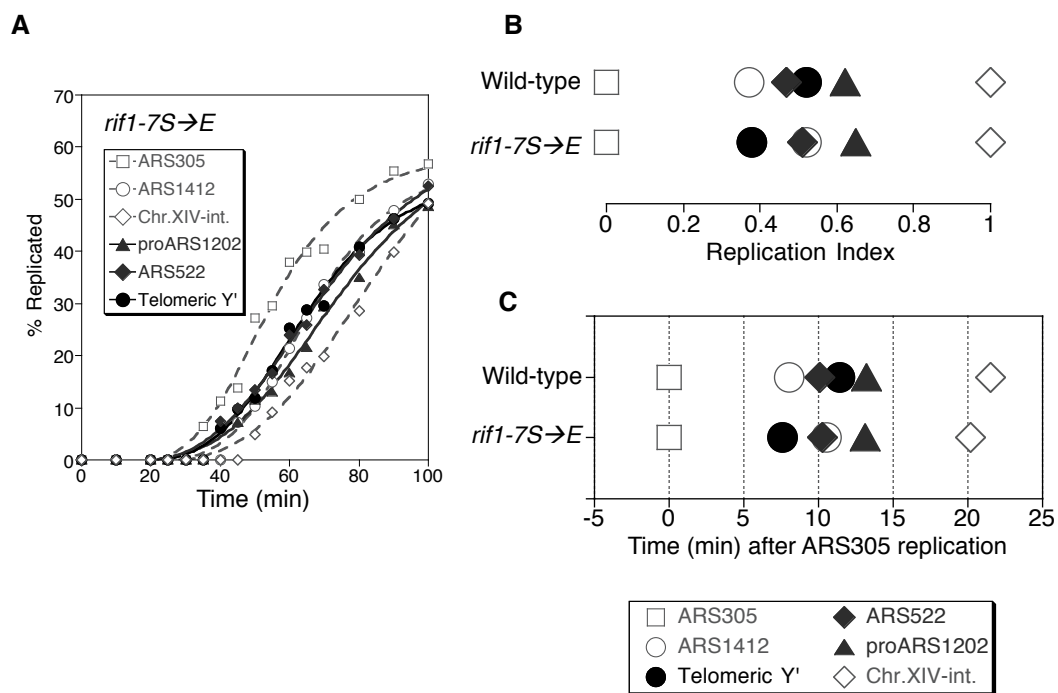

**Figure S12.** In the *rif1-7S→E* mutant telomere replication time is not advanced relative to ARS1412.

Supplement: Figure S12 — In the rif1-7S→E mutant telomere replication time is not advanced relative to ARS1412. (A) Replication program of rif1-7S→E, released from an α-factor block at 30°C. Sequences analyzed are as in Fig. 1. (B) Replication indices from rif1-7S→E experiment shown in A, along with values from wild-type experiment from Fig. 1B&C. (C) Replication times (from experiments in A) plotted relative to the replication time of early origin ARS305 (set to time = 0 min). Strains used are ASY73 (rif1-7S→E) and BB14-3a (wild-type). (PDF) [file pgen.1004691.s014.pdf]
