# Supplementary material for: At Short Telomeres Tel1 Directs Early Replication and Phosphorylates Rif1
Source: PLoS Genet. 2014 Oct 16;10(10):e1004691. doi: 10.1371/journal.pgen.1004691 (PMC4199499; doi:10.1371/journal.pgen.1004691)
Supplement: Figure S4 — Tel1 is required for efficient activation in hydroxyurea of the ARS700.5 origin neighboring an induced short telomere. (A) Evaluating the efficiency of HO cutting used to generate a single short telomere. Cartoon of inducible short telomere construct is shown in main Fig. 2A. Cells were grown in 2% Raffinose (Asynchronous), arrested with α factor in 4% Galactose (lanes 2–4 and 10–12) and released into S phase in the presence of HU (lanes 5–8 and 13–16). XmnI-digested DNA samples were probed for the 5′ part of ADE2 (see Fig. 2A). Percentage cutting is indicated, prior to release and 120 min after release into HU. Asterisk indicates ARS1412 fragment, also probed in this experiment. (B) Quantification of the bubble arc in ARS700.5 relative to loading as assessed by the intensity of the ‘1N spot’ of non-replicating DNA. Boxes illustrate the area used for intensity quantification. (C) Table showing relative intensity values of the bubble arc and 1N spots for ARS700.5, ARS305 and ARS1412. Bubble arc values were extracted from long 2D gel exposures and 1N spot values extracted from short 2D gel exposures, to maintain phosphorimager signal linearity. After normalization for loading, the reduction in origin activation of ARS700.5 was 4.8-fold in the tel1Δ strain relative to TEL1. Origin activation levels were in contrast hardly affected for early origin ARS305 and late origin ARS1412. Gels used for quantification shown in Fig. 2C. Strains used are SMKY10 (TEL1) and SMKY13 (tel1Δ). (PDF) [file pgen.1004691.s006.pdf]

A

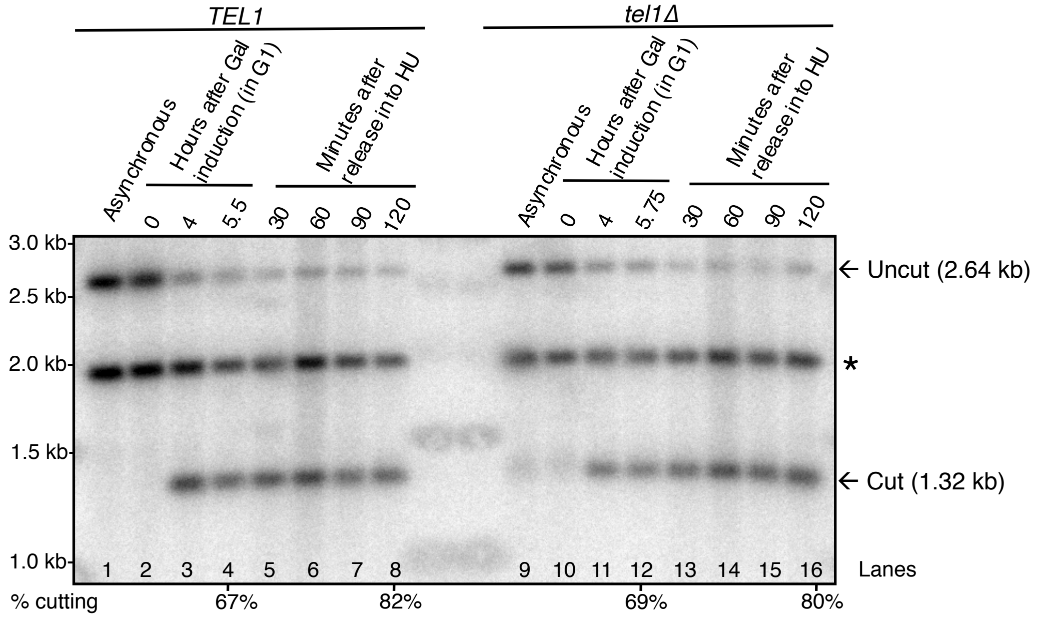

B

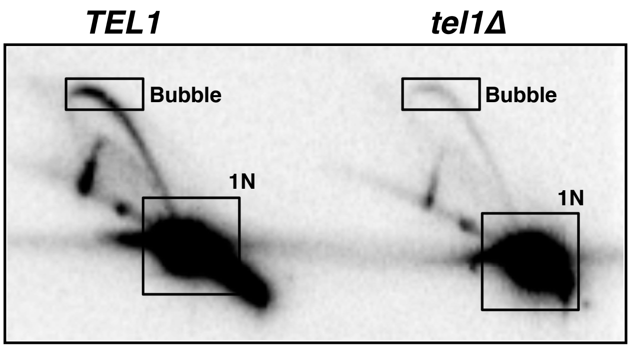

C

	1N spot (= Loading)		Bubble		Fold change in *tel1Δ* (normalized to loading)
	TEL1	***tel1Δ***	***TEL1***	***tel1Δ***	
**ARS700.5**	120,999	101,607	22,529	3,977	0.21
**ARS305**	129,389	180,569	21,983	27,864	0.91
**ARS1412**	586,753	671,186	1,216	1,279	0.92

Figure S4. Tel1 is required for efficient activation in hydroxyurea of the ARS700.5 origin neighboring an induced short telomere.
