# Supplementary material for: At Short Telomeres Tel1 Directs Early Replication and Phosphorylates Rif1
Source: PLoS Genet. 2014 Oct 16;10(10):e1004691. doi: 10.1371/journal.pgen.1004691 (PMC4199499; doi:10.1371/journal.pgen.1004691)
Supplement: Figure S8 — Abundance of a longer Rif1 peptide, phosphorylated at Serine-1308, is decreased in the absence of Tel1. (A) Plots shows relative levels of S-1308 phosphorylated peptide [KVDS(ph)QDIQVPATQGM(ox)KEPPSSIQISSQISAK] in yku70Δ (Light-labeled) and yku70Δ tel1Δ (Heavy-labeled) strains. This is a longer peptide encompassing the same sequence as the peptide in Fig. 5A, containing a lysine not cleaved during the trypsin digestion. (B) MS spectrum of the same peptide [KVDS(ph)QDIQVPATQGM(ox)KEPPSSIQISSQISAK] comparing relative abundance in yku70Δ (R0K0-labeled) and yku70Δtel1Δ (R10K8-labeled). (C) MS spectrum comparing abundance of the non-phosphorylated form of the peptide KVDSQDIQVPATQGM(ox)KEPPSSIQISSQISAK in yku70Δ (R0K0-labeled) and yku70Δ tel1Δ (R10K8-labeled). (PDF) [file pgen.1004691.s010.pdf]

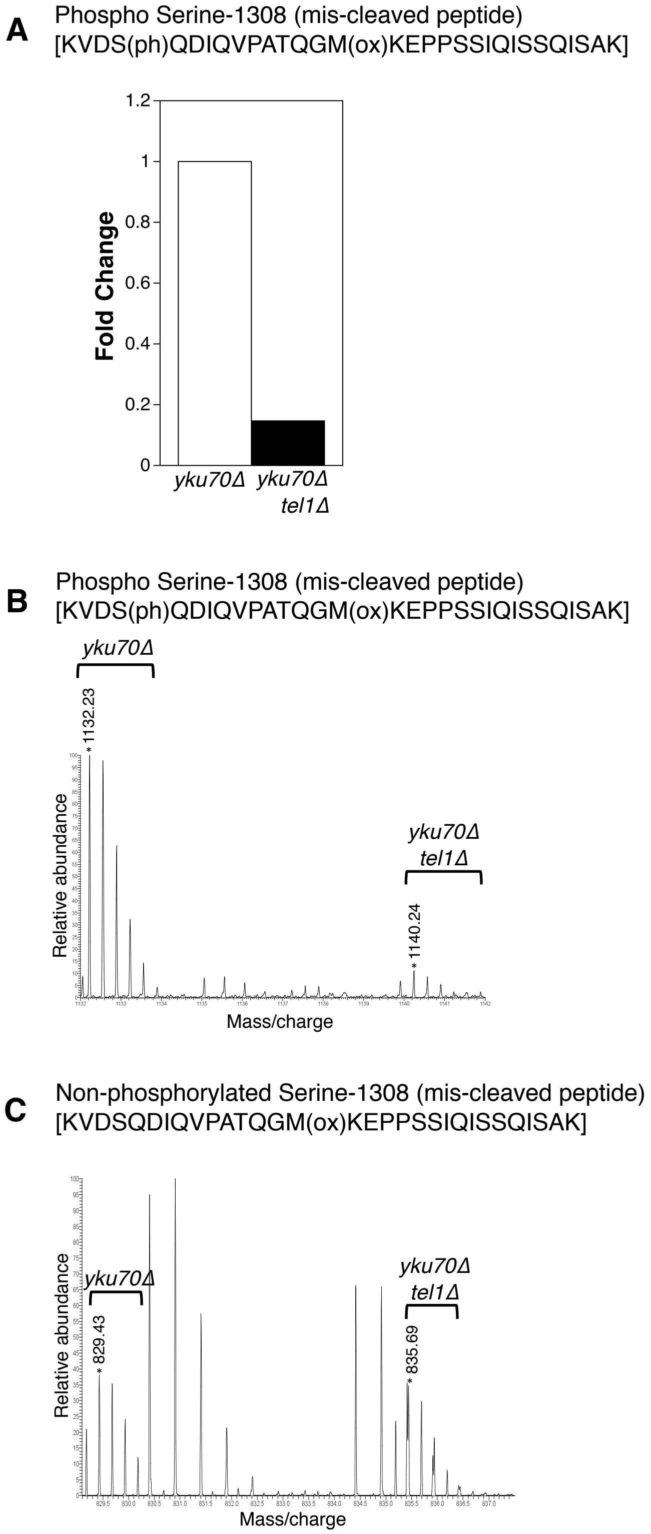

Figure S8. Abundance of a longer Rif1 peptide, phosphorylated at Serine-1308, is decreased in the absence of Tel1
