# Supplementary material for: At Short Telomeres Tel1 Directs Early Replication and Phosphorylates Rif1
Source: PLoS Genet. 2014 Oct 16;10(10):e1004691. doi: 10.1371/journal.pgen.1004691 (PMC4199499; doi:10.1371/journal.pgen.1004691)
Supplement: Figure S10 — Replication times show that the non-phosphorylatable Rif1 does not delay the early replication of yku70Δ short telomeres. Replication times (from experiments in Fig. 6B), plotted relative to the replication time of early origin ARS305 (set to time = 0 min), along with values from wild-type and yku70Δ experiments from Fig. 1 and S1). Strains used are ASY76 (rif1-7S→A yku70Δ), BB14-3a (wild-type) and AW99 (yku70Δ). (PDF) [file pgen.1004691.s012.pdf]

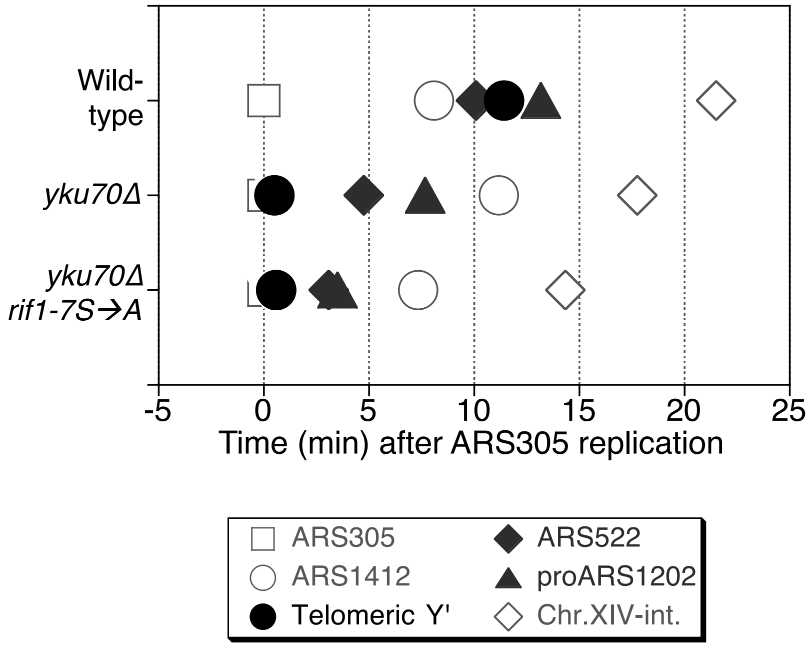

Figure S10. Replication times show that non-phosphorylatable Rif1 does not delay the early replication of *yku70Δ* short telomeres.
