# Supplementary material for: At Short Telomeres Tel1 Directs Early Replication and Phosphorylates Rif1
Source: PLoS Genet. 2014 Oct 16;10(10):e1004691. doi: 10.1371/journal.pgen.1004691 (PMC4199499; doi:10.1371/journal.pgen.1004691)
Supplement: Figure S11 — Non-phosphorylatable Rif1 does not affect telomeric replication times in YKU70 strain background. (A) Replication program of rif1-7S→A, released from an α-factor block at 30°C. Sequences analyzed are as in Fig. 1. (B) Replication indices from rif1-7S→A experiment shown in A, along with values from wild-type experiment from Fig. 1B&C. (C) Replication times (from experiments in A) plotted relative to the replication time of early origin ARS305 (set to time = 0 min). Strains used are ASY69 (rif1-7S→A) and BB14-3a (wild-type). (PDF) [file pgen.1004691.s013.pdf]

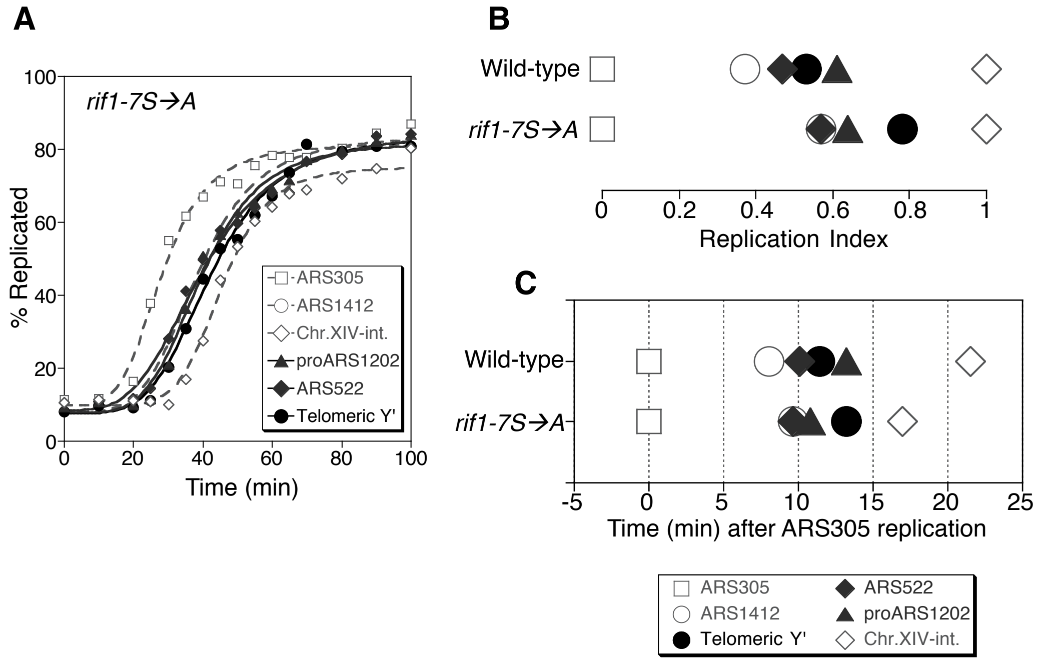

Figure S11. Non- phosphorylatable Rif1 does not affect telomeric replication times in *YKU70* strain background.
