# Supplementary material for: At Short Telomeres Tel1 Directs Early Replication and Phosphorylates Rif1
Source: PLoS Genet. 2014 Oct 16;10(10):e1004691. doi: 10.1371/journal.pgen.1004691 (PMC4199499; doi:10.1371/journal.pgen.1004691)
Supplement: Table S1 — Yeast strains. Yeast strains used in this study are listed along with their source and the figures where used. (DOC) [file pgen.1004691.s015.doc]

**At short telomeres Tel1 directs early replication and phosphorylates Rif1**

Akila Sridhar, Sylwia Kedziora and Anne D. Donaldson*

Table S1. Yeast strains used in this study

| **Strain**  **name** | **Genotype** | **Strain Background** | **References and figures where used** |
| --- | --- | --- | --- |
| BB14-3a | *MATa* *ura3-52 his6 trp1-289 leu2-3,112 bar1∆* | A364a | [52]  Fig.1, 3A&C, 6A&C, S1, S2, S3, S6, S10, S11B&C and S12B&C |
| AW99 | BB14-3a *yku70∆::URA3* | A364a | [8]  Fig.1, 6A&C, S1, S2, S3 and S10 |
| HYLS44 | BB14-3a *rif1∆::LEU2* | A364a | [4]  Fig.3, 4A, 6A and S6 |
| ASY5 | BB14-3a *tel1∆::CaURA3* | A364a | This Study  Fig.1, 3A&C, 6A, S1, S2, S3 and S6 |
| ASY13 | BB14-3a *tel1∆::CaURA3 yku70∆::TRP1* | A364a | This Study  Fig.1, S1, S2 and S3 |
| ASY14 | BB14-3a *tel1∆::CaURA3 rif1∆::LEU2* | A364a | This Study  Fig.3 and S6 |
| YSM20 | *MATa ade2-1 can1-100 his3-11,15 leu2-3,112 trp1-1 ura3-1, RIF1-13Myc::HIS3MX6* | W303 *RAD5* | David Shore  Fig.4C |
| ASY17 | *YSM20 yku70∆::TRP1* | W303 *RAD5* | This Study  Fig.4C |
| Y0000 (=BY4741) | MATa; *his3∆1*; *leu2∆0*; *met15∆0*; *ura3∆0* | BY4741 | Euroscarf  Fig. S2 |
| Y03114 | BY4741 *tel1∆::KanMX* | BY4741 | Euroscarf  Fig.S2 |
| Y00870 | BY4741 *yku70∆::KanMX4* | BY4741 | Euroscarf  Fig. 4A and S2 |
| Y07170 | BY4741 *rif1∆::KanMX* | BY4741 | Euroscarf  Used to generate strain used in Fig. S6B |
| SHY201 | *MAT*a *his3∆1 leu2∆0 lys2∆0 ura3∆0 arg4∆::natMX4* | BY4741 | [46]  Fig.4A, B(i)&(ii) |
| ASY25 | *SHY201*  *RIF1-13Myc::HIS3MX6* | BY4741 | This Study  Fig. 4A, B(i)&(ii), D-G and S7 |

| ASY30 | *SHY201*  *RIF1-13Myc::HIS3MX6 yku70∆::URA3* | BY4741 | This Study  Fig.4A, B(ii), D-G, 5, S7 and S8 |
| --- | --- | --- | --- |
| ASY46 | *SHY201*  *RIF1-13Myc::HIS3MX6 yku70∆::URA3 tel1∆::KanMX* | BY4741 | This Study  Fig.5 and S8 |
| ASY51 | *BB14-3a rif1-scd∆::URA3 (also referred to as rif1∆scd)* | A364a | This Study  Fig.6A |
| ASY69 | *BB14-3a rif1-7S🡪A* | A364a | This Study  Fig. 6A and S11 |
| ASY73 | *BB14-3a rif1-7S🡪E* | A364a | This Study  Fig.6A and S12 |
| ASY76 | *BB14-3a rif1-7S🡪A yku70∆::URA3* | A364a | This Study  Fig. 6 and S10 |
| ASY78 | *BB14-3a rif1-7S🡪E yku70∆::URA3* | A364a | This Study  Fig. 6A |
| ASY81 | *BB14-3a RIF1-7S🡪S* | A364a | This Study  Fig.6A |
| YAB1410 | *ade2-1 his3-11,15 leu2-3,112 trp1-1 ura3-1 can1-100 RAD5 TelVII-L::TG80-HO-CA250* | W303 Rad5 | Alessandro Bianchi  Fig. 2A |
| YAB1356 | *ade2-1 his3-11,15 leu2-3,112 trp1-1 ura3-1 can1-100 RAD5 TelVII-L::TG250-HO* | W303 Rad5 | Alessandro Bianchi  Fig. S5 |
| SMKY10 | *YAB1410 ura3-1∆::URA3* | W303 Rad5 | This study  Fig. 2, S4 and S5 |
| SMKY13 | *YAB1410 ura3-1∆::URA3 tel1∆::KanMX* | W303 Rad5 | This study  Fig. 2 and S4 |
| SMKY15 | *SMKY10 rif1∆::KanMX* | W303 Rad5 | This study  Fig. S6B |
