# Supplementary material for: At Short Telomeres Tel1 Directs Early Replication and Phosphorylates Rif1
Source: PLoS Genet. 2014 Oct 16;10(10):e1004691. doi: 10.1371/journal.pgen.1004691 (PMC4199499; doi:10.1371/journal.pgen.1004691)
Supplement: Text S1 — Supplementary experimental procedures. Text file with in-detail procedures for strain construction, Rif1 immunoprecipitation, and mass spectrometry and data analyses. (DOC) [file pgen.1004691.s016.doc]

**At short telomeres Tel1 directs early replication and phosphorylates Rif1**

Akila Sridhar, Sylwia Kedziora and Anne D. Donaldson*

**Supplemental experimental procedures**

**Yeast strain constructions**

*S. cerevisiae* strains used are listed in Supplemental Table S1. Gene knockouts were made using standard PCR-based insertion and homologous recombination techniques , with correct integration of markers confirmed by PCR. In strains ASY5, ASY13 and ASY14, *TEL1* was knocked out with *Candida albicans URA3* (*CaURA3*) amplified from plasmid pAG60 . To generate a *yku70∆::TRP1* allele, the *TRP1* marker was amplified from plasmid YDp-W using primers with *YKU70* flanking sequence. The *rif1∆::LEU2* and *yku70∆::URA3* alleles were generated similarly using plasmids YDp-L and YDp-U.

Inducible HO cut strains (YAB1410 and YAB1356) were kindly gifted by Alessandro Bianchi. In these strains, the HO cut site construct contains an *ADE2* marker and is inserted into the *ADH4* locus ~18 kb from the left end of chromosome VII. YAB1356 (inducible long telomere) has an HO endonuclease cut site with long telomeric sequence (TG250) on the centromere-proximal side. YAB1410 (inducible short telomere) has an HO endonuclease cut site flanked by a short telomeric sequence (TG80) on the centromere-proximal side and a long telomeric sequence (TG250) on the telomere-proximal side. For technical reasons, YAB1410 was converted to Ura^+^ by transforming a wild-type *URA3* gene into the native mutated *ura3-1* locus, to generate SMKY10. *TEL1* was knocked out with *KanMX* amplified from Y03114 (from Euroscarf), to generate SMKY13. *RIF1* was similarly knocked out with *KanMX* amplified from Y07170 (from Euroscarf), to generate SMKY15.

The Myc-tagged *RIF1* allele in strain YSM20 (a gift from David Shore) was a standard PCR-based insertion based on homologous recombination . The C-terminal tag is inserted immediately after the last amino acid (Asparagine), using a *13-Myc::HIS3MX6* amplified from plasmid pFA6-13Myc. The tag was confirmed by sequencing. Primer sequences are available on request. For SILAC analysis, the 13Myc allele was amplified from YSM20 and inserted into SILAC-compatible strain SHY201.

To construct Rif1 phospho-site mutants, strain ASY51 was made in which the *RIF1* sequence from nucleotides 3903-4724 (corresponding to amino acids 1301-1574 in the protein sequence) was replaced with *URA3* to delete the cluster of seven S/TQ sites, making a *rif1∆scd*::*URA3* ('*rif1∆scd'*) allele. An 822bp fragment containing either seven Serine to Alanine substitutions (*rif1-7S🡪A*) or seven Serine to Glutamic acid substitutions (*rif1-7S🡪*E) was synthesized (Dundee Cell Products). Each fragment was transformed into ASY51, selecting against *URA3* using 5-FOA. The mutagenized alleles created were confirmed by sequencing. A similar method was used to reconstruct wild-type *RIF1* (i.e. inserting the wild-type sequence back into *rif1∆scd*) generating a *RIF1-7S🡪S* strain, as a control to ensure recovery of normal telomere length following the mutagenesis procedure.

**Rif1 Immunoprecipitation Procedure**

To immunoprecipitate Rif1 for mass spectrometry, 1-2 litres of mid-log phase cells (cell density ~1x10^10^ cells/litre) were collected and washed twice in 20mM HEPES-KOH (pH 7.9), 1M Sorbitol. Cells were washed again in 100mM HEPES-KOH (pH 7.9), 0.8M Sorbitol, 1% TritonX-100, 1M NaCl and 3mM MgCl_2_ and the cell pellet was weighed. The pellet was then resuspended in a volume three times the pellet weight of Lysis buffer [100mM HEPES-KOH (pH 7.9), 3mM Mgcl_2_, 1M NaCl, 1mM DTT, 2mM β-glycerophosphate, 2mM Sodium Fluoride, 1mM PMSF, 1X Roche inhibitor cocktail (cOmplete EDTA-free, Roche, 04693132001), 1% Protease inhibitor (Sigma, P2815), 1X Phosphatase inhibitor cocktail (PhosSTOP, Roche, 04906845001), and 1% Triton X-100 (to solubilize most of the Rif1 cellular pool)]. The cell suspension was dripped into liquid nitrogen, to form yeast ‘popcorn’. The ‘popcorn’ was then milled under liquid nitrogen using a Freezer mill (SPEX SamplePrep 6750 freezer/mill) with the following instrument settings: Pre-cool-2 min, 7 cycles of Run-2 min and Cool – 2min, at Rate 14. The lysed cell suspension (Whole Cell Extract) was then clarified by centrifuging twice at 16,000xg. The supernatant was then diluted four-fold with Lysis buffer lacking NaCl, to obtain a final concentration of 250mM NaCl and a volume of 15-20 ml. Immunoprecipitation was carried out with 80μg Rabbit anti-Myc antibody (ab9106, Abcam) coupled to 500μl Dynabeads Protein G (Invitrogen, 10003D), rotating overnight at 4°C. Beads were then washed with 4 times with 1 ml of 100mM HEPES-KOH (pH 7.9), 200mM NaCl, 3mM MgCl_2_, 0.1% NP-40, 1mM DTT, 2mM β-glycerophosphate, 2mM Sodium Fluoride, 1mM PMSF, 1X Roche inhibitor cocktail (cOmplete EDTA-free, Roche, 04693132001), 1% Protease inhibitor (Sigma, P2815) and 1X Phosphatase inhibitor cocktail (PhosSTOP, Roche, 04906845001). Proteins were then eluted from the beads by boiling with 1X SDS sample buffer (Invitrogen) containing 5% 2-mercaptoethanol. Protein concentrations were estimated using the RCDC protein assay kit (Bio-rad).

**Mass Spectrometry Analysis**

Trypsin-digested peptides were separated using an Ultimate 3000 RSLC (Thermo Scientific) nanoflow LC system. On average 0.5 µg was loaded with a constant flow of 5 µl/min onto an Acclaim PepMap100 nanoViper C18 trap column (100 µm inner-diameter, 2cm; Thermo Scientific). After trap enrichment, peptides were eluted onto an Acclaim PepMap RSLC nanoViper, C18 column (75 µm, 15 cm; ThermoScientific) with a linear gradient of 2–40% solvent B (80% acetonitrile with 0.08% formic acid) over 65 min with a constant flow of 300 nl/min. The HPLC system was coupled to a linear ion trap Orbitrap hybrid mass spectrometer (LTQ-Orbitrap Velos, Thermo Scientific) via a nanoelectrospray ion source (Thermo Scientific). The spray voltage was set to 1.2 kV, and the temperature of the heated capillary was set to 250 °C. Full-scan MS survey spectra (*m*/*z* 335–1800) in profile mode were acquired in the Orbitrap with a resolution of 60,000 after accumulation of 1,000,000 ions. The fifteen most intense peptide ions from the preview scan in the Orbitrap were fragmented by collision-induced dissociation (normalized collision energy, 35%; activation Q, 0.250; and activation time, 10 ms) in the LTQ after the accumulation of 10,000 ions. Maximal filling times were 1,000 ms for the full scans and 150 ms for the MS/MS scans. Precursor ion charge state screening was enabled along with multistage activation for neutral loss ions for phosphorylation site analysis, and all unassigned charge states as well as singly charged species were rejected. The lock mass option was enabled for survey scans to improve mass accuracy . Data were acquired using the Xcalibur software, and peptide SILAC ratios extracted using MaxQuant software version 1.3.0.5 as below, with phosphorylation as a possible modification.

**Quantification and bioinformatics analysis**

#### The raw mass spectrometric data files obtained for each experiment were collated into a single quantitated data set using MaxQuant (version 1.3.0.5) and the Andromeda search engine software . Enzyme specificity was set to that of trypsin, allowing for cleavage N-terminal to proline residues and between aspartic acid and proline residues. Other parameters used were: (i) variable modifications, methionine oxidation, protein N-acetylation, gln🡪pyro-glu, Phospho (STY); (ii) fixed modifications, cysteine carbamidomethylation; (iii) database: target-decoy human MaxQuant (ipi.HUMAN.v3.68); (iv) heavy labels: R10K8; (v) MS/MS tolerance: FTMS- 10ppm, ITMS- 0.6 Da; (vi) maximum peptide length, 6; (vii) maximum missed cleavages, 2; (viii) maximum of labeled amino acids, 3; and (ix) false discovery rate, 1%. Peptide ratios were calculated for each arginine- and/or lysine-containing peptide as the peak area of labeled arginine/lysine divided by the peak area of non-labeled arginine/lysine for each single-scan mass spectrum. Peptide ratios for all arginine- and lysine-containing peptides sequenced for each protein were averaged. Data is normalized using 1/median ratio value for each identified protein group per labeled sample.

**References**

1. Longtine MS, McKenzie A, 3rd, Demarini DJ, Shah NG, Wach A, et al. (1998) Additional modules for versatile and economical PCR-based gene deletion and modification in *Saccharomyces cerevisiae*. Yeast 14: 953-961.

2. Goldstein AL, Pan X, McCusker JH (1999) Heterologous URA3MX cassettes for gene replacement in *Saccharomyces cerevisiae*. Yeast 15: 507-511.

3. Berben G, Dumont J, Gilliquet V, Bolle PA, Hilger F (1991) The YDp plasmids: a uniform set of vectors bearing versatile gene disruption cassettes for *Saccharomyces cerevisiae*. Yeast 7: 475-477.

4. Olsen JV, de Godoy LMF, Li GQ, Macek B, Mortensen P, et al. (2005) Parts per million mass accuracy on an orbitrap mass spectrometer via lock mass injection into a C-trap. Molecular & Cellular Proteomics 4: 2010-2021.

5. Cox J, Mann M (2008) MaxQuant enables high peptide identification rates, individualized p.p.b.-range mass accuracies and proteome-wide protein quantification. Nat Biotechnol 26: 1367-1372.

6. Cox J, Neuhauser N, Michalski A, Scheltema RA, Olsen JV, et al. (2011) Andromeda: A Peptide Search Engine Integrated into the MaxQuant Environment. Journal of Proteome Research 10: 1794-1805.
